# Targeted lung *Lactobacillus johnsonii* intervention alleviates virus-induced fibrosis post-HCT through PD-L1/PD-1 signaling

**DOI:** 10.1126/sciadv.adw4654

**Published:** 2026-01-01

**Authors:** Joshua B. Perkins, Keerthikka Ravi, Chunfang Guo, Gina J. Oh, Bellmary Garcia Rodriguez, Chin-Ning Chen, Selga I. Jansons, Faye Sun, Stephen J. Gurczynski, Jason B. Weinberg, Gary B. Huffnagle, David N. O’Dwyer, Bethany B. Moore, Xiaofeng Zhou

**Affiliations:** ^1^ Department of Microbiology and Immunology, University of Michigan, Ann Arbor, MI, USA.; ^2^ Department of Molecular, Cellular, and Developmental Biology, University of Michigan, Ann Arbor, MI, USA.; ^3^ Division of Infectious Diseases, Department of Pediatrics, University of Michigan, Ann Arbor, MI, USA.; ^4^ Mary H. Weiser Food Allergy Center, University of Michigan, Ann Arbor, MI, USA.; ^5^ Division of Pulmonary & Critical Care Medicine, Department of Internal Medicine, University of Michigan, Ann Arbor, MI, USA.

## Abstract

Our study highlights the decrease of *Lactobacillus johnsonii* in the lungs following hematopoietic cell transplantation (HCT) and its immunomodulatory effects in attenuating post-HCT pulmonary complications. Introducing live or heat-killed *L. johnsonii* into the lungs of HCT mice significantly reduced gammaherpesvirus-induced lung inflammation and fibrosis. This protective effect was mediated in part by the up-regulation of PD-L1 on dendritic cells, which in turn dampened the production of the inflammatory cytokine IL-17A by T helper 17 cells post-HCT. *L. johnsonii* also reduced *Tgfb1* expression in lung macrophages. These anti-fibrotic effects of heat-killed *L. johnsonii* were absent in PD-1–deficient mice, highlighting the role of PD-L1/PD-1 signaling. Further analysis showed that dendritic cells uniquely recognized *L. johnsonii* and increased PD-L1 expression via TLR1/2- and TLR9-MyD88 pathways. Our findings suggest that heat-killed lactobacilli could serve as a safe postbiotic therapy to moderate immune responses and reduce lung inflammation and fibrosis post-HCT, offering a strategy for managing transplant-related lung complications.

## Teaser

Delivering a beneficial bacterium to the lungs helps reduce virus-induced lung scarring after bone marrow transplantation.

## INTRODUCTION

The lung microbiome is characterized by low biomass and a constant state of flux; however, various features of the bacterial communities in the lung microbiome have been found to correlate with pulmonary health and pathology (*1*). Alterations of lung microbiota diversity and composition have been noted in lung diseases such as idiopathic pulmonary fibrosis (IPF) (*2*, *3*), chronic obstructive pulmonary disease, acute respiratory distress syndrome, HIV, lung cancer, and asthma (*4*). Conversely, studies in mouse models have demonstrated that in healthy mice, the immune tone of the lung, as measured by baseline levels of interleukin-1 alpha (IL-1α) and IL-4, is closely correlated with the diversity and community composition of the lung, but not the gut microbiome. These findings highlight the importance of the local lung microbiome in influencing pulmonary immune responses (*5*). As our understanding of how the lung microbiome interacts with the immune system in health and disease advances, the possibility of developing microbiome-based therapeutics aimed at modulating immune responses in the lungs emerges. However, there are notable challenges to overcome before these can be applied clinically. These include the identification of precise microbial targets for specific lung conditions, elucidating the mechanisms by which these targets influence lung health and immune tone and determining the most effective formulations and routes for therapeutic delivery (*1*, *6*).

Medical procedures frequently disrupt the host’s microbiome, which in turn can be linked to adverse clinical outcomes. This is particularly evident in the context of hematopoietic cell transplantation (HCT), where both the procedure itself and the multiple exposures to antibiotic treatment that are common in patients with HCT can substantially alter the gut microbiome (*7*) and the lung microbiome (*8*). Pulmonary complications, affecting up to 60% of allogeneic HCT recipients (*9*) and 25% of autologous HCT recipients (*10*), are often preceded by dysbiosis in the lung and gut microbiome, suggesting an important relationship between microbiome and lung health following transplantation (*11*).

Idiopathic pneumonia syndrome (IPS) is a serious “noninfectious” pulmonary complication of HCT that is characterized by pneumonitis, lung injury, and often fibrosis, leading to irreversible morbidity and mortality (*12*). The specific causative factors of IPS are incompletely defined; however, more sensitive testing has revealed that occult respiratory pathogens can be identified in 50% of IPS cases [most commonly human herpesvirus 6 (HHV-6)], and patients with occult pathogens present have higher mortality (*13*). We also demonstrated a marked association between a history of herpesvirus reactivation shortly after transplant, such as HHV-6 and Epstein-Barr virus (EBV), and IPS development in human HCT recipients (*14*). In preclinical studies, we found that acute infection with murine gammaherpesvirus 68 (MHV-68), a mouse virus with similarities to the human gammaherpesvirus EBV, following syngeneic (syn) HCT in mice, induces an IL-17–dependent inflammatory pneumonitis and subsequent progression to fibrotic lung disease, mirroring key elements of human IPS pathology (*15*–*17*). Clinical studies have also found that IL-6, a key cytokine driving T helper 17 cell (T_H_17) differentiation, is markedly elevated in patients with IPS following allogeneic HCT. The local IL-6 secretion promotes expansion of donor T_H_17 cells within lung tissue and drives IL-17A–mediated pulmonary injury (*18*).

Intriguingly, a notable dysbiosis occurs within the lung bacterial community post-HCT, particularly a significant depletion of the *Lactobacillus* genus (*8*). Similarly, in bronchoalveolar lavage fluid from human HCT recipients diagnosed with IPS, the family Lactobacillaceae members were nearly undetectable (*8*). Many *Lactobacillus* species/strains are recognized as probiotics and have the potential to modulate specific mucosal immune system functions (*19*). However, the immunomodulatory effects and therapeutical potential of lactobacilli in alleviating inflammation and pulmonary fibrosis following HCT have not yet been investigated.

In this study, we found that the strain *Lactobacillus johnsonii* XZ17 (GenBank accession number CP151183.1) (*20*) is prevalent in the lungs of healthy non-HCT mice but diminished following HCT. We found that the intranasal administration of either live or heat-killed (HK) *L. johnsonii* XZ17 to HCT mice mitigates inflammation and pulmonary fibrosis. Treatment with HK *L. johnsonii* XZ17 increased programmed death-ligand 1 (PD-L1) expression on dendritic cells (DCs) and programmed cell death protein-1 (PD-1) on T_H_17 cells, which in turn suppressed the production of IL-17A, a key mediator of pneumonitis and fibrosis in HCT mice. DCs recognized *L. johnsonii* and up-regulate their PD-L1 expression via Toll-like receptor 2 (TLR2)– and TLR9-myeloid differentiation primary response 88 (MyD88) pathways. The ability to mitigate pulmonary fibrosis in HCT mice was unique to *L. johnsonii* and not seen with *Ligilactobacillus murinus* or methicillin-resistant *Staphylococcus aureus* (MRSA). In addition, HK *L. johnsonii* XZ17 exerted anti-inflammatory effects by inhibiting the expression of pro-inflammatory cytokines such as *Il6*, *Il18*, and profibrotic *Tgfb1* in lung macrophages in a PD-1–dependent manner. The use of heat-inactivated *L. johnsonii* offers enhanced safety for immunotherapy in immunocompromised patients. Overall, our findings have identified a specific microbial agent that acts in a PD-L1/PD-1–dependent manner as an immunomodulator to potentially offer a safe and effective strategy to reduce pulmonary complications post-HCT.

## RESULTS

### Mouse syn-HCT diminishes *L. johnsonii* in the lung microbiome

**Fig. 1.**
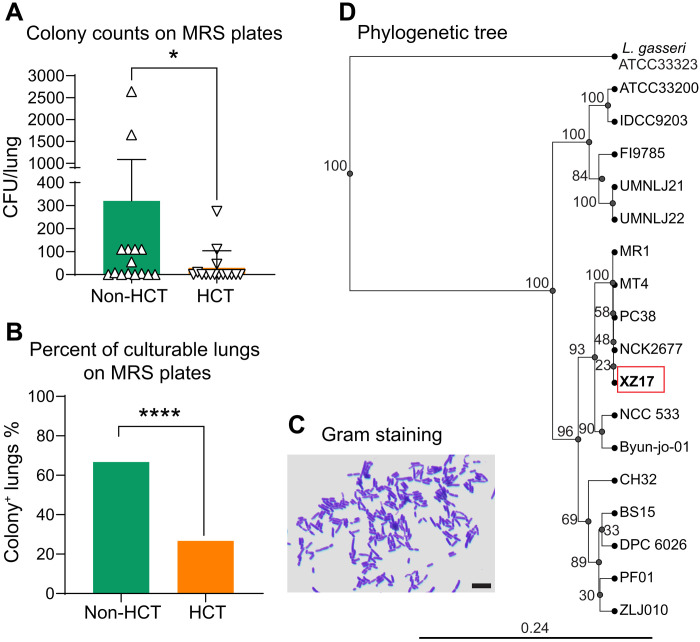
*L. johnsonii* is diminished following syn-HCT in mouse lungs. (**A**) Lungs were isolated from 15 C57BL/6J mice 5 weeks after receiving syn-HCT or from age-matched, healthy non-HCT controls. Each lung was homogenized and subsequently plated onto MRS agar plates for the quantification of *Lactobacillus* colony-forming units (CFUs). The CFUs per lung homogenate were counted 24 hours after plating. (**B**) Percentage of mouse lungs with positive *Lactobacillus* cultures (*n* = 15 per group). (**C**) Gram staining of bacterial colonies cultured from the lungs of a non-HCT C57BL/6J mouse. Scale bar, 5 μm. (**D**) Pairwise whole genome sequence ANI score–based phylogenetic tree showing the relationship of the *L. johnsonii* XZ17 strain (red rectangle) to 17 other *L. johnsonii* sequences sourced from the National Center for Biotechnology Information database, along with the *L. johnsonii* type strain ATCC 33200. *Lactobacillus gasseri* ATCC 33323, a closely related species is used as the out-group for the analysis. The phylogenetic tree was built using hierarchal clustering of the Euclidean distance matrix of pairwise ANI scores. The number on the nodes represents the bootstrap value. The bar at the bottom provides the scale of the branch length. For (A), data are expressed as means ± SEM, with *P < 0.05 as determined by the Mann-Whitney *U* test. For (B), ****P < 0.0001 was determined by Fisher’s exact test.

HCT itself and the subsequent medications and infectious complications profoundly alter the lung microbiome (*8*, *21*). We previously developed a mouse model in which syn-HCT followed by MHV-68 infection led to severe pneumonitis and pulmonary fibrosis (*16*, *17*). We have shown that, similar to its effects in humans, HCT modifies the lung microbiome composition in mice, and a subsequent MHV-68 infection further reduces community diversity (*8*). We also found that the relative abundance of members of the genus *Lactobacillus* was significantly reduced in the lungs of mice following HCT (*8*). To verify these results, we cultured lung homogenates from non-HCT or HCT mice on de Man-Rogosa-Sharpe (MRS) agar, a selective medium that promotes the growth of lactobacilli (*22*). Lungs from non-HCT mice harbored a higher number of culturable lactobacilli compared to those from HCT-treated mice (Fig. 1A), Among those tested lungs, 10 of the 15 (66.7%) non-HCT mouse lung samples yielded lactobacillus colonies on MRS agar, whereas only 4 of the 15 (26.7%) samples from HCT recipients did so (Fig. 1B). These colonies were morphologically consistent and composed of Gram-positive rods (Fig. 1C). Eight colonies were randomly selected for molecular analysis. We performed bidirectional sequencing of the 16*S* ribosomal RNA (rRNA) gene using the D88 forward and E94 reverse degenerate primers (*23*). The sequencing resulted in high-quality reads with an approximate length of 1170 base pairs, revealing that the sequences of all isolates were identical. The sequences showed complete identity with the known 16*S* rRNA gene sequence of *L. johnsonii* (*24*). Thus, we have identified a bacterial species that is significantly diminished in the lungs after HCT in mice.

We further sequenced the whole genome of a selected colony, designated as *L. johnsonii* strain XZ17, and submitted the sequence to GenBank (accession number CP151183.1) as detailed by Ravi *et al.* (*20*). The genome of strain XZ17 comprises 1,948,040 base pairs with a guanine and cytosine (GC) content of 34.64%, and it contains 1780 predicted protein-coding genes. Phylogenetic analysis revealed that the genome of XZ17 shares a high degree of similarity with other mouse isolates from the Jackson Laboratory, including MR1, MT4, PC38, and NCK2677, exhibiting an average nucleotide identity (ANI) of 99.9%. XZ17 also has a notable similarity of 98.4% with the human probiotic isolate NCC 533, which clusters with all mouse isolates in the phylogenetic tree (Fig. 1D). Notably, the related MR1 strain (*25*), which becomes enriched in the cecum of BALB/cJ mice after administration with house dust collected from homes with indoor/outdoor dogs, has been shown to enhance airway immune defense against allergens and respiratory syncytial virus (RSV) infection when administered intragastrically (*26*, *27*).

### Administration of *L. johnsonii* mitigates pulmonary fibrosis in herpesvirus-infected HCT mice

*L. johnsonii* can reduce inflammation and mucogenic responses caused by RSV infection (*26*–*28*), and as shown above, it is significantly diminished after HCT (Fig. 1, B and C). On the basis of this evidence, we chose to reintroduce the *L. johnsonii* XZ17 strain into the lungs of HCT mice. To determine the colonization potential of the XZ17 strain in the lungs of HCT mice, 5 × 10^5^ colony-forming units (CFU) XZ17 were administered intranasally to HCT mice. This low dose is presumed to be physiologically relevant, as it mirrors the typically low biomass of the lung microbiome compared to that of the gut microbiome (*29*). Our results indicated an approximately two-log reduction of XZ17 CFUs 1 day postinstillation, and by day 3, virtually no culturable XZ17 remained (fig. S1A). Live/dead bacterial staining (fig. S1B) revealed that despite the absence of culturable XZ17 in the lungs 3 days after instillation, a small proportion of the XZ17 bacteria remained in a viable but nonculturable state (fig. S1C). These results imply that the HCT lung environment is inhospitable for *L. johnsonii* colonization, likely owing to conditions such as elevated oxidative stress and insufficient nutrient availability. This result also suggests that lactobacilli normally found in the lungs of healthy mice are likely transient visitors, routinely dispersed from the upper respiratory tract, rather than permanent inhabitants.

**Fig. 2.**
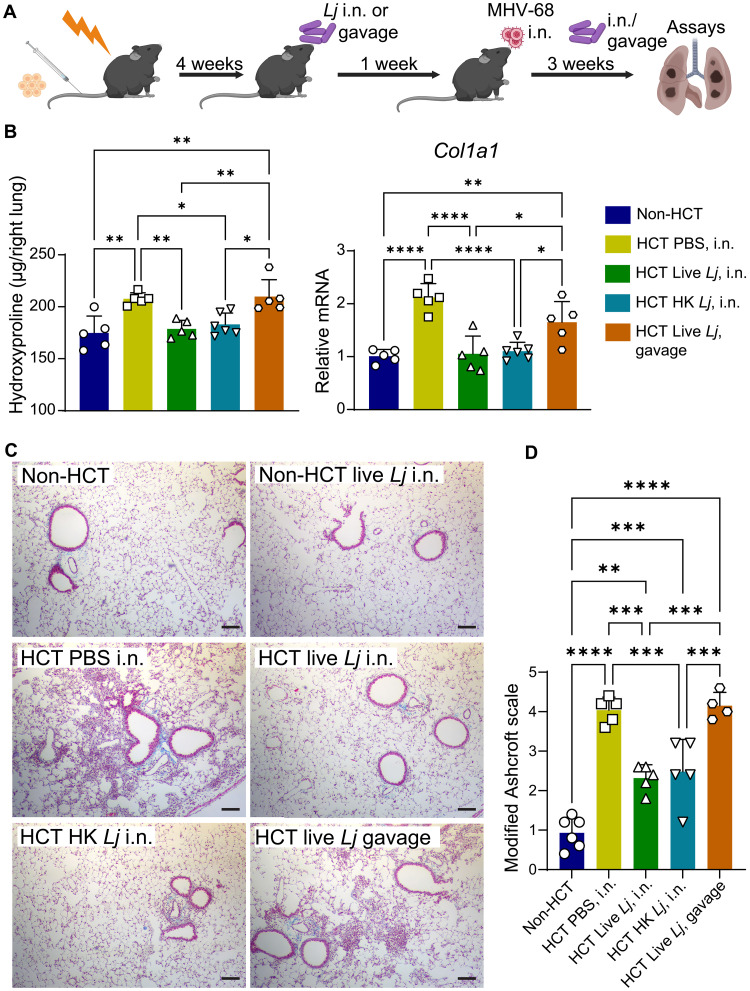
Intranasal administration of live or HK *L. johnsonii* XZ17 reduces inflammation and pulmonary fibrosis in HCT mice infected with MHV-68. (**A**) Schematic of the experimental design [created with BioRender. X. Zhou (2025) https://BioRender.com/7ifs8rv]. C57BL/6J mice (*n* = 5 to 6 per group) were lethally irradiated before transplantation with 5 × 10^6^ bone marrow cells from donor C57BL/6J mice. At 4 weeks posttransplantation, either 5 × 10^5^ CFU of live *L. johnsonii* XZ17 (Live *Lj*) or an equivalent amount of HK *L. johnsonii* XZ17 (HK *Lj*) was administered intranasally to recipient mice at intervals of 2 to 3 days. Alternatively, mice were given 4 × 10^7^ CFU of live XZ17 via oral gavage at the same schedule. One week after the initial intranasal or oral administration of *L. johnsonii* XZ17, all mice, including the non-HCT controls, were infected intranasally with 5 × 10^4^ PFU of MHV-68, followed by continuous administration of XZ17, and lung tissues were collected at 21 dpi. (**B**) Collagen content in the right lung was assessed by the hydroxyproline assay (left), and relative mRNA expression levels of *Col1a1* in the left lung tissue was measured by quantitative polymerase chain reaction (qPCR) (right). All samples were from MHV-68–infected mice. (**C**) Representative images of lung tissue sections of MHV-68–infected mice stained with Masson’s Trichrome. Scale bars, 100 μm. Collagen is stained blue. (**D**) Modified Ashcroft scoring for fibrosis evaluation. Data are presented as means ± SEM. Statistical significance is indicated by *P < 0.05, **P < 0.01, ***P < 0.001, and ****P < 0.0001, as determined by one-way analysis of variance (ANOVA) with Tukey’s multiple comparisons test. Results are representative three independent experiments. i.n., intranasal.

To assess the impact of the XZ17 strain on the lungs of HCT mice, we administered 5 × 10^5^ CFU of live XZ17 or an equivalent quantity of HK XZ17 intranasally to HCT mice every 2 or 3 days, beginning 4 weeks after HCT and 7 days before MHV-68 infection and continuing for a duration of 3 weeks postinfection (Fig. 2A). HK XZ17 was prepared by incubating the bacteria in a 70°C water bath for 15 min. For the oral route, we administered live 4 × 10^7^ CFU XZ17 via oral gavage following the same schedule as intranasal administration. Previous studies have demonstrated that oral administration at this dosage of *L. johnsonii* can alleviate pathology associated with RSV infection (*26*, *27*). Results showed that intranasal administration of either live or HK XZ17 significantly reduced collagen content and collagen type I alpha 1 chain (*Col1a1*) expression in the lungs of HCT mice at 21 days postinfection (dpi) with MHV-68, as compared to levels measured in mock-inoculated controls (Fig. 2B). In contrast, high-dose oral administration of live XZ17 did not significantly attenuate pulmonary fibrosis in MHV-68–infected HCT mice (Fig. 2B). The histological examination of the lungs using Masson’s Trichrome staining supported the differential impact of intranasal versus oral administration on collagen accumulation (Fig. 2, C and D). The fact that both live and HK XZ17 alleviated virus-induced pulmonary fibrosis in HCT mice implies the structural components of the bacteria, rather than secreted soluble factors such as metabolites, were sufficient for effects on fibrosis. To determine whether other HK Gram-positive bacteria could also prevent fibrosis in MHV-68–infected mice post-HCT, we tested another *Lactobacillus* species, *L. murinus*, which is abundant in the murine gut (*30*), and an unrelated Gram-positive bacterium, MRSA. Unlike HK *Lj*, neither of these HK bacteria attenuated fibrosis following infection in HCT mice (fig. S2). Thus, the therapeutic effects observed with HK *Lj* may be unique to this bacterial species. Since HK *L. johnsonii* offers the benefit of mitigating pulmonary fibrosis without the risk of causing infection in immunocompromised HCT recipients, subsequent studies focus on deciphering the mechanisms underlying the effects of the HK *L. johnsonii* XZ17 strain, henceforth referred to as HK *Lj* administration.

### HK *L. johnsonii* administration exerts anti-inflammatory effects on macrophages and T_H_17 cells

**Fig. 3.**
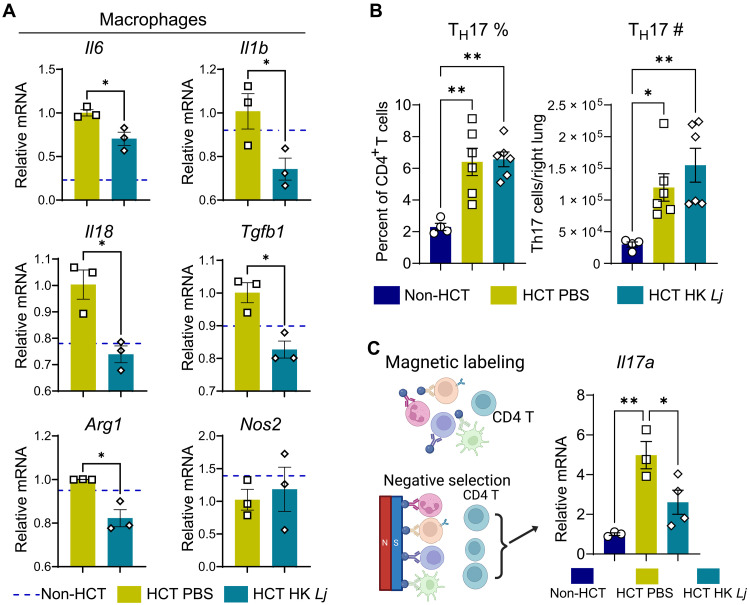
Intranasal administration of HK *L. johnsonii* XZ17 reduces inflammation in macrophages and T_H_17 cells at 7 dpi with MHV-68. Non-HCT C57BL/6J mice (*n* = 4) and HCT mice with mock or HK *Lj* administration (*n* = 6 per group) were intranasally infected with MHV-68, and lung tissues were harvested at 7 dpi. (**A**) Relative mRNA expression levels of a variety of cytokines in lung macrophages at 7 dpi with MHV-68. Macrophages were isolated from pooled single-cell suspensions of two lungs using microbeads conjugated with anti-F4/80 antibodies, and qPCR was used to assess relative mRNA expression levels. The dashed lines represent expression levels of each gene in lung macrophages from non-HCT mice infected with MHV-68. (**B**) Flow cytometry was used to measure the proportion of T_H_17 cells (CD45^+^CD3^+^CD4^+^IL-17^+^) within the CD4^+^ T cell population (left, *n* = 4 to 6) and absolute T_H_17 cell numbers (right) in the right lung. (**C**) Schematic of the negative selection process used to enrich for untouched lung CD4^+^ T cells [left, created in BioRender. X. Zhou (2025) https://BioRender.com/6y28nml]; the qPCR analysis of *Il-17a* mRNA expression in isolated CD4^+^ T cells (*n* = 3 to 4 mice, right). Data are presented as means ± SEM. For (A), statistical significance is denoted by *P < 0.05, calculated using unpaired two-tailed Student’s *t* tests. For (B) and (C), statistical significance is denoted by *P < 0.05 and **P < 0.01, determined by one-way ANOVA with Tukey’s multiple comparisons test. Results are representative of two independent experiments.

Pulmonary fibrosis often develops following an inflammatory response to initial lung injury (*31*). To determine whether intranasal administration of HK *Lj* mitigates pulmonary fibrosis by inhibiting inflammatory responses in the lungs of HCT mice infected with MHV-68, we conducted flow cytometry analyses on single-cell suspensions of lung immune cells prepared at 7 dpi after lung perfusion. This perfusion protocol effectively removes most intravascular DCs and macrophages, as confirmed by anti-CD45.2 intravenous staining (fig. S3A). However, the CD4^+^ T cell compartment consists of ~60% intraparenchymal and 40% intravascular cells after perfusion (fig. S3A). No significant differences in the proportions of CD4^+^ T cell subsets, specifically T_H_1, T_H_17, and regulatory T cells (T_reg_ cells), between the intraparenchymal and intravascular fractions were detected across experimental groups (fig. S3, B to D). The gating strategy used is presented in fig. S4. At 7 dpi, the total lung cells were comparable between non-HCT and HCT mice regardless of the administration of HK *Lj* (fig. S5A). The percentage of CD45^+^ cells was higher in HCT mice than in non-HCT mice, and HK *Lj* treatment did not change the frequency or numbers of CD45^+^ cells (fig. S5A). HK *Lj* administration did not significantly alter the relative or absolute numbers of many immune cell types, including T cells, neutrophils, natural killer cells, resident and inflammatory monocytes (rMono and iMono), interstitial macrophages (IM), type II conventional DCs (cDC2), monocyte-derived DCs (MoDCs), and eosinophils (fig. S5, B and C). Tissue-resident alveolar macrophages (TRAM) were diminished in the lungs following HCT and infection, and administration with HK *Lj* did not restore their numbers. HK *Lj* administration led to a decrease in the numbers of B cells and cDC1 in the lungs of HCT mice at 7 dpi (fig. S5, B and C). Intriguingly, HK *Lj* administration increased recruitment of monocyte-derived alveolar macrophages (MoAMs), although HK *Lj* administration did not increase the expression of *Ccl2*, a chemokine that typically mediates the recruitment of monocytes and macrophages in the lung (fig. S6A) (*32*). We observed a trend toward decreases in the mRNA levels of the inflammatory cytokine genes *Il6* and *Il1b* following HK *Lj* administration in the whole lung (fig. S6, B and C). Considering that macrophages are key producers of inflammatory cytokines, and MoAMs are critically involved in the development of pulmonary fibrosis (*32*, *33*), the total lung RNA may obscure significant alterations in macrophage transcripts. Therefore, we enriched macrophages from lung single-cell suspensions using microbeads conjugated to anti-F4/80 antibodies to further assess the effects of HK *Lj* on inflammatory responses. We observed a significant reduction in the mRNA expression levels of inflammatory classically activated macrophages (M1-like) cytokines (*Il6*, *Il1b*, and *Il18*) in macrophages from the lungs of HCT mice treated with HK *Lj*, while the classic M1 marker nitric oxide synthase 2 (*Nos2*) remained unchanged (Fig. 3A). In addition, we found significantly decreased mRNA levels of alternatively activated macrophage (M2)-like markers transforming growth factor–β1 (*Tgfb1*) and Arginase 1 (*Arg1*) in lung macrophages (Fig. 3A). Notably, activated TGF-β1 is a key contributor to the onset of pulmonary fibrosis (*17*, *34*). Our findings align with a reduction in M2-like profibrotic activity (*35*, *36*) and further suggest that the remaining M1-polarized macrophages may be functionally less active. Despite these changes in innate immunity, HK *Lj* administration neither enhanced nor impaired viral clearance (fig. S6D). Collectively, these results indicate that although HK *Lj* administration promotes the recruitment of MoAMs to the lungs, it also exerts nuanced, suppressive effects on both M1-like and M2-like macrophage phenotypes.

We have established in previous research that lung T_H_17 cells (CD45^+^CD3^+^CD4^+^IL-17^+^) are increased in HCT mice after MHV-68 infection and that IL-17A is essential for the development of pulmonary fibrosis in this viral-induced HCT fibrosis model (*15*, *16*, *37*). However, to our surprise, the percentage and absolute numbers of T_H_17 cells in lungs at 7 dpi were not diminished by HK *Lj* administration, as determined by phorbol 12-myristate 13-acetate (PMA)/ionomycin stimulation, intracellular anti–mouse IL-17 antibody staining and flow cytometry analysis (Fig. 3B). To investigate whether HK *Lj* administration could suppress T_H_17 cell expression of *Il17a* in HCT mice, we measured their *Il17a* mRNA expression in CD4^+^ T cells enriched from lungs (Fig. 3C). We found a significant decrease in *Il17a* mRNA expression in lung CD4^+^ T cells isolated from HK *Lj*-supplemented HCT mice at 7dpi (Fig. 3C). Thus, while HK *Lj* administration did not inhibit the polarization of T_H_17 cells in the lungs of HCT mice, it did reduce *Il17a* expression in those cells.

### HK *L. johnsonii* administration enhances PD-L1 expression on cDC2s, MoDCs, and alveolar epithelial cells

The observed decrease in T_H_17 cell expression of *Il17a* in HCT mice that received HK *Lj* administration does not appear to be attributable to increased percentages of T_reg_ cell, since we did not observe an effect of HK *Lj* administration on the proportion of conventional T_reg_ cells or retinoic acid–related orphan receptor gamma t–positive (RORγt^+^) T_reg_ cells in HCT mice (fig. S7, A and B). This is in contrasts with a recent report in which an enhancement of immunosuppressive RORγt^+^ T_reg_ cells in the lungs was seen in naïve mice after intranasal administration of a *L. murinus* strain (*38*).

**Fig. 4.**
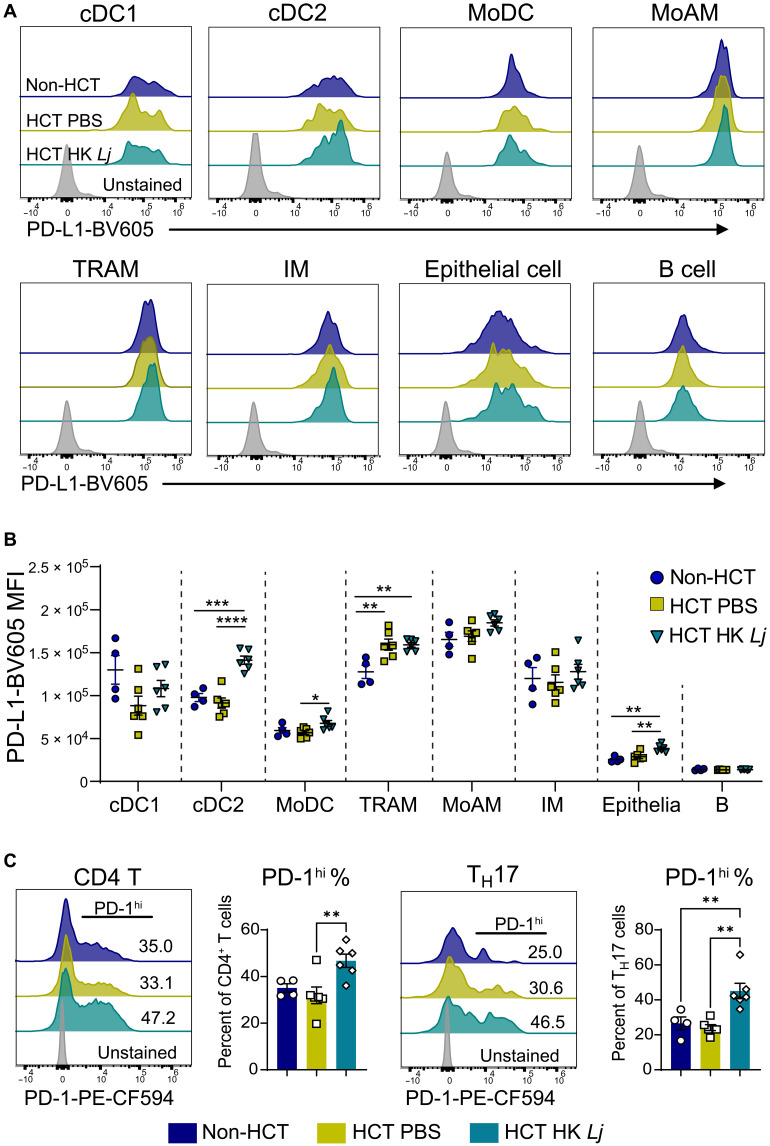
Intranasal administration of HK *L. johnsonii* XZ17 augments PD-L1 expression on the cell surfaces on cDC2s, MoDCs, and alveolar epithelial cells, and PD-1 expression on T_H_17 cells. Non-HCT C57BL/6J mice (*n* = 4) and HCT mice with mock or HK *Lj* administration (*n* = 6 per group) were euthanized at 7 dpi, and their lung single-cell suspensions were prepared for flow cytometry analysis. (**A**) Representative flow cytometry histograms illustrating PD-L1 expression on MHC II^+^ cells, including DCs, macrophages, epithelial cells, and B cells, at 7 dpi with MHV-68 (*n* = 4 to 6). (**B**) Mean fluorescence intensity (MFI) quantification of PD-L1 expression on DCs, macrophages, epithelial cells, and B cells at 7 dpi (*n* = 4 to 6). (**C**) Left: Representative histograms displaying PD-1 expression on CD4^+^ T cells accompanied by percentages of PD-1^hi^ CD4^+^ T cells (*n* = 4 to 6). Right: Representative histograms presenting PD-1 expression on T_H_17 cells accompanied by percentages of PD-1^hi^ T_H_17 cells (*n* = 4 to 6). For (B) and (C), data are presented as means ± SEM, and statistical significance is represented by *P < 0.05, **P < 0.01, ***P < 0.001, and ****P < 0.0001, as determined by one-way ANOVA with Tukey’s multiple comparisons test. Results are representative of two independent experiments. MoAM, monocyte-derived alveolar macrophage; TRAM, tissue-resident alveolar macrophage; IM, interstitial macrophage; PE, phycoerythrin;

We reasoned that HK *Lj* administration may enhance the expression of PD-L1 on the surface of antigen-presenting cells, which could in turn suppress T_H_17 cell activity via the PD-L1/PD-1 signaling pathway (*39*).We found that HK *Lj* administration increased PD-L1 expression on the surface of cDC2s, MoDCs, and major histocompatibility complex (MHC) II^+^ EpCAM^+^ alveolar epithelial cells (which express PD-L1 at much lower levels). In contrast, cell surface PD-L1 levels on macrophages were not further increased by HK *Lj* administration at 7 dpi (Fig. 4, A and B). Since lung cDC2s often acquire expression of cluster of differentiation 64 (CD64) and the MAR-1 antigen (recognized by an anti-FcεRI antibody), which are markers also shared with MoDCs (*40*), we further used CD26, CD64, and MAR-1 to distinguish cDC2s from MoDCs (fig. S8, A to C). As expected, there is some overlap in the expression of CD64 and MAR-1 between cDC2 and MoDC populations (fig. S8D). However, even with this refined gating strategy, we observed a significant increase in PD-L1 expression on both cDC2 and MoDC populations following HK *Lj* treatment (fig. S8E). Given that cDC2s play a critical role in directing the polarization of T_H_17 cells (*15*, *41*), our findings imply that HK *Lj* administration may target and inhibit T_H_17 cell activities through cDC2s. Although no decrease of total CD4^+^ or CD8^+^ T cell numbers were observed in HK *Lj*–supplemented HCT mice (fig. S9, A and B), the percentage of PD-1^hi^ cells in the CD4^+^ T cell and T_H_17 cell compartments at 7 dpi was increased in the lungs of HCT mice supplemented with HK *Lj* (Fig. 4C). We detected a similar increase in the percentage of PD-1^hi^ CD8^+^ T cells (fig. S9C). The HK *Lj* administration also up-regulates Tim-3, another checkpoint receptor and late marker of T cell exhaustion (*42*), on CD4^+^, CD8^+^, and T_H_17 cells. Notably, many T cells were positive for either PD-1 or Tim-3 alone (fig. S10), suggesting that HK *Lj* may regulate T cell checkpoint activity and exhaustion through multiple, distinct pathways. Together, HK *Lj* administration increased the expression of PD-L1 on DCs, which potentially suppresses the expression of *Il17a* in T_H_17 cells in HCT lungs by augmenting PD-L1/PD-1 signaling.

### HK *L. johnsonii*–treated BMDCs inhibit iT_H_17 cells via PD-L1/PD-1 signaling

**Fig. 5.**
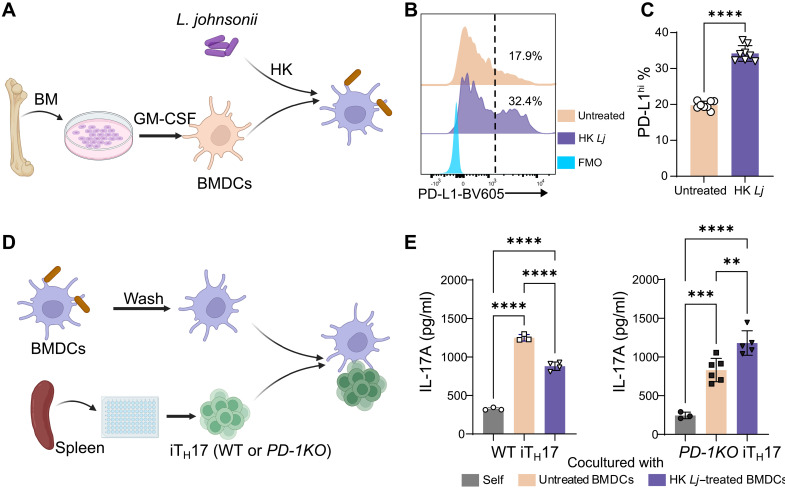
HK *L. johnsonii* XZ17 enhances PD-L1 expression on BMDCs and suppresses IL-17A production in iT_H_17 cells via PD-L1/PD-1 signaling. (**A**) A diagram depicting the procedure to derive BMDCs from bone marrow cells and their subsequent coculture with HK *Lj*. (**B**) Flow cytometry histograms showing PD-L1 expression on BMDCs. Cells to the right of the dashed line are high expressers of PD-L1 (PD-L1^hi^), with numbers indicating the percentage of these cells. (**C**) Proportion of PD-L1^hi^ BMDCs (*n* = 8 per group). (**D**) Diagram illustrating the generation of iT_H_17 cells and their coculture with HK *Lj*–treated BMDCs. (**E**) Enzyme-linked immunosorbent assay (ELISA) was used to measure IL-17A concentrations in coculture supernatants. The left panel shows cocultures with BMDCs and WT iT_H_17 cells; the right panel shows cocultures with BMDCs and PD-1KO iT_H_17 cells. *N* = 3 to 6 culture supernatants. Data are expressed as means ± SEM. For (C), statistical significance is denoted by ****P < 0.0001, determined using unpaired two-tailed Student’s *t* tests. For (E), statistical significance is indicated by **P < 0.01, ***P < 0.001, and ****P < 0.0001 using one-way ANOVA with Tukey’s multiple comparisons test. The depicted results are representative of two independent experiments. GM-CSF, granulocyte-macrophage colony-stimulating factor; FMO, fluorescence minus one. (A) and (D) created in BioRender. X. Zhou (2025) https://BioRender.com/rlk4ehc.

The in vivo studies described above indicate that HK *Lj* administration leads to the up-regulation of PD-L1 on the surface of DCs in HCT mice. To test if HK *Lj* directly enhances the DC surface expression of PD-L1, we generated bone marrow–derived dendritic cells (BMDCs) from a C57BL/6J male mouse [wild type (WT)]. Granulocyte-macrophage colony-stimulating factor (GM-CSF) was used to induce the differentiation of bone marrow progenitor cells into DCs with phenotypic similarities to cDC2s and MoDCs (*43*). These BMDCs were cultured with HK *Lj* at a 1:2 ratio for 24 hours (Fig. 5A). A significant increase in PD-L1 expression on BMDCs was observed following exposure to HK *Lj* (Fig. 5, B and C, and fig. S11). To evaluate whether the up-regulation of PD-L1 on BMDC represses T_H_17 cell secretion of IL-17A, we cocultured untreated or HK *Lj*–treated BMDCs with induced T_H_17 (iT_H_17) cells, which were differentiated from naïve CD4^+^ T cells from the spleens of WT or PD-1 knockout (PD-1KO) mice (Fig. 5D) (*44*). For iT_H_17 cells derived from WT mice, a notable reduction in IL-17A secretion occurred when cocultured with HK *Lj*–treated BMDCs, compared to iT_H_17 cells cocultured with untreated BMDCs. Conversely, iT_H_17 cells derived from PD-1KO mice did not exhibit suppressed IL-17A production following exposure to HK *Lj*–treated BMDCs. Instead, IL-17A production was significantly increased when PD-1KO iT_H_17 cells were cocultured with HK *Lj*–treated BMDCs (Fig. 5E). These results strongly suggest that HK *Lj* exposure of BMDCs suppresses iT_H_17 cell production of IL-17A through engagement of the PD-L1/PD-1 signaling axis.

### PD-1 is required for HK *L. johnsonii*’s immunoregulation after HCT and infection

Building on our previous findings that HK *Lj* increases PD-L1 expression on antigen-presenting cells (Fig. 4, A and B) and reduces IL-17A production from iT_H_17 cells via the PD-L1/PD-1 axis in vitro (Fig. 5), we hypothesized that PD-L1/PD-1 signaling mediates HK *Lj*’s immunomodulatory effects to suppress IL-17A in vivo. To test this hypothesis, we developed PD-1KO HCT mice, wherein bone marrow cells from PD-1KO mice were transplanted into irradiated syn recipients. In these PD-1KO mice, immune cells are unresponsive to PD-L1 signaling from PD-L1–expressing cells. We analyzed immune responses in C57BL/6J (WT) non-HCT, WT HCT, and PD-1KO HCT mice, each treated with either HK *Lj* or phosphate-buffered saline (PBS) and infected with MHV-68 with lung harvest at 7 dpi.

**Fig. 6.**
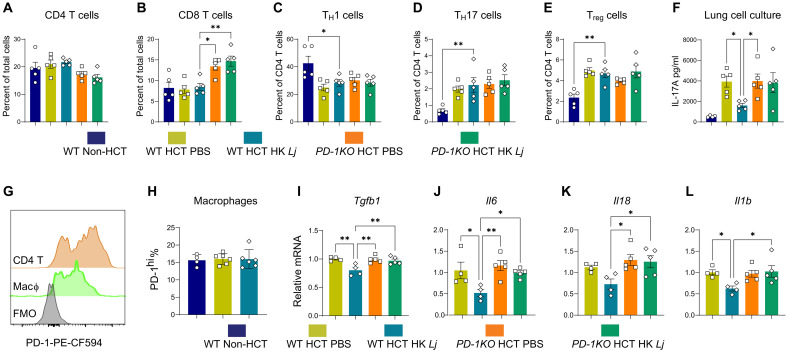
PD-1 is required for HK *L. johnsonii*–mediated suppression of pathogenic T_H_17 cell and macrophage responses in MHV-68–infected HCT. (**A** to **E**) Flow cytometry analysis of lung T lymphocytes at 7 days post–MHV-68 infection in C57BL/6J (WT) non-HCT, WT HCT, and PD-1KO HCT mice treated with PBS or HK *Lj*. Shown are the percentages of total CD4^+^ T cells (A), CD8^+^ T cells (B), and CD4^+^ T cell subsets: T_H_1 (C), T_H_17 (D), and T_reg_ (E). (**F**) IL-17A concentration in culture supernatants following 36-hour ex vivo culture of lung leukocytes. (**G**) Representative flow cytometry histogram of PD-1 expression on CD4^+^ T cells and macrophages (CD45^+^CD64^+^MHC II^+^) from a WT HCT mouse treated with HK *Lj*, with FMO control. (**H**) Frequency of PD-1^hi^ macrophages among total macrophages. (**I** to **L**) Relative mRNA expression of *Tgfb1* (I), *Il6* (J), *Il18* (K), and *Il1b* (L) in lung macrophages sorted with anti-F4/80 microbeads, determined by qPCR. Each data point represents an individual mouse; bars indicate means ± SEM. Statistical significance: *P < 0.05 and **P < 0.01 (one-way ANOVA with Tukey’s multiple comparisons), compared to the WT HCT + HK *Lj* group. Comprehensive pairwise comparisons between all groups (one-way ANOVA with Tukey’s multiple comparisons) are presented in fig. S12. The depicted results are representative of two independent experiments.

Flow cytometry showed no significant differences in the proportion of total CD4^+^ T cells among groups at 7dpi (Fig. 6A and fig. S12A). However, PD-1KO HCT mice displayed a significant increase in CD8^+^ T cells compared to WT HCT controls, which was unaffected by HK *Lj* treatment (Fig. 6B and fig. S12B). CD4^+^ T cell subset frequencies—including T_H_1 (Fig. 6C and fig. S12C), T_H_17 (Fig. 6D and fig. S12D), and T_reg_ (Fig. 6E and fig. S12E)—remained unchanged by HK *Lj* administration in both WT and PD-1KO HCT mice. Functionally, HK *Lj* treatment significantly reduced IL-17A production by lung leukocytes of WT HCT mice [enzyme-linked immunosorbent assay (ELISA) assay, 36-hour culture], whereas this suppression was absent in PD-1KO HCT mice (Fig. 6F and fig. S12F). In a model of antibody-mediated PD-1 blockade, the neutralization of PD-1 in WT HCT mice led to increased *Il17a* mRNA expression, but similarly, HK *Lj* supplementation did not suppress *Il17a* levels (fig. S13A). In addition, although a higher proportion of T_H_17 cells persisted after PD-1 blockade, HK *Lj* had no effect on T_H_17 differentiation in this context (fig. S13B). These data underscore the requirement for PD-L1/PD-1 signaling in HK *Lj*–mediated suppression of IL-17A production.

Since macrophages also express surface PD-1, albeit at lower levels than CD4^+^ T cells (Fig. 6G), we hypothesized that PD-1 may play a role in regulating macrophage activity during HK *Lj* treatment. We did not observe any changes in the proportion of PD-1^hi^ macrophages (CD45^+^CD64^+^MHC II^+^) following HK *Lj* administration (Fig. 6H and fig. S12H). Strikingly, the ability of HK *Lj* to suppress the expression of the profibrotic factor *Tgfb1* (Fig. 6I) and inflammatory cytokines (*Il6*, *Il18*, and *Il1b*; Fig. 6, J to L, and fig. S12, J to L) in macrophages was abolished in PD-1KO HCT mice, indicating a similar dependence on PD-1 for regulatory effects in macrophages. Collectively, these findings demonstrate that HK *L. johnsonii* suppresses pathogenic IL-17A production by T_H_17 cells, as well as profibrogenic and inflammatory cytokine expression by macrophages, through a PD-1–dependent mechanism.

### HK *L. johnsonii* reduces fibrosis in MHV-68–infected HCT lungs via PD-L1/PD-1 signaling

**Fig. 7.**
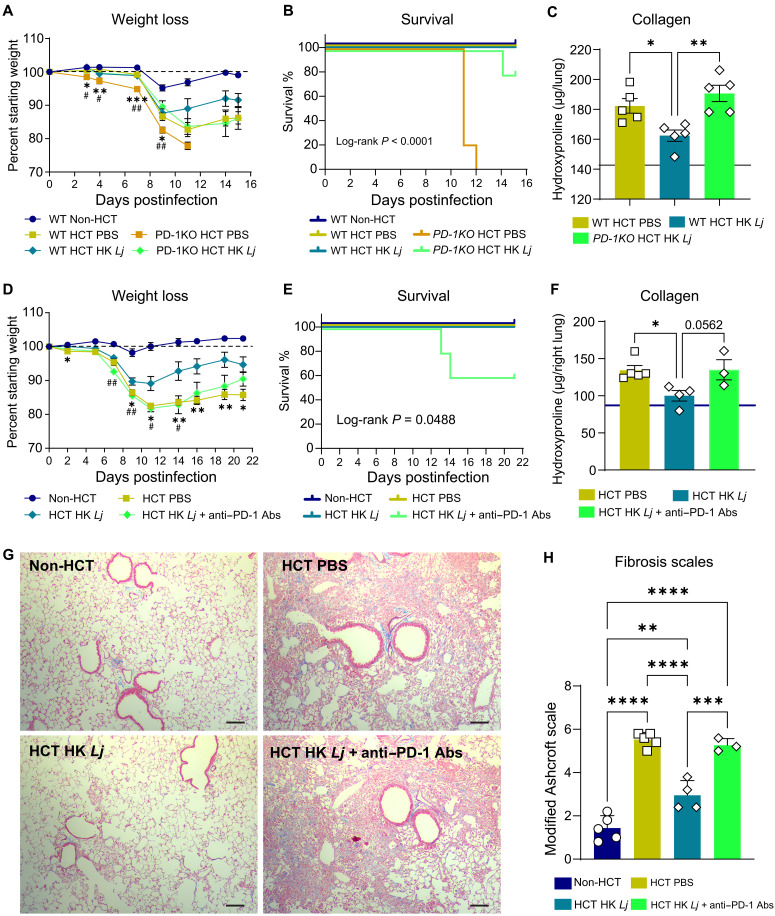
The PD-L1/PD-1 signaling axis is required for HK *L. johnsonii* XZ17 to alleviate inflammation and pulmonary fibrosis in HCT mice after MHV-68 infection. (**A** to **C**) Effects of HK *Lj* administration on HCT mice reconstituted with PD-1KO or WT bone marrow. C57BL/6J mice (*n* = 5 per group) received bone marrow transplants from either WT or PD-1KO mice, followed by intranasal HK *Lj* or PBS beginning 4 weeks posttransplant and recurring every 2 to 3 days. Mice were subsequently infected intranasally with MHV-68 and monitored until 15 dpi or moribund. Data are representative of three independent experiments. (A) Weight loss after infection. (B) Survival curves. (C) Collagen content in whole lungs by hydroxyproline assay. (Note that the group of PD-1KO HCT mice without HK *Lj* was not included due to mortality at ~11 dpi.). (**D** to **H**) Effects of HK *Lj* in PD-1–neutralized HCT mice. WT HCT mice were given anti–PD-1 antibodies (Abs) or PBS (*n* = 3 to 5 per group) intraperitoneally from 2 dpi. (D) Weight loss after MHV-68 infection. (E) Survival curves. (F) Collagen quantification in right lungs. (G) Masson’s Trichrome staining of left lung sections showing fibrosis (collagen, blue; scale bars, 100 μm). (H) Ashcroft fibrosis scores. Data are means ± SEM. For (A) and (D), statistical significance in body weight changes was assessed by unpaired two-tailed Student’s *t* test; *P < 0.05, **P < 0.01, and ***P < 0.001 (HCT PBS versus HCT HK *Lj*); #*P* < 0.05 and ##*P* < 0.01 (PD-1–deficient HCT PBS versus PD-1–deficient HCT HK *Lj*). For (C), (F), and (H), significance was determined by one-way ANOVA with Tukey’s multiple comparisons test; *P < 0.05, **P < 0.01, ***P < 0.001, and ****P < 0.0001.

Given that PD-1 mediates the immunomodulatory effects of HK *Lj* during acute MHV-68 infection, characterized by the suppression of profibrogenic factors IL-17A and TGF-β and several inflammatory cytokines (Fig. 6), we hypothesized that the reduction of pulmonary fibrosis in MHV-68–infected HCT mice following intranasal HK *Lj* administration is dependent on activation of the PD-L1/PD-1 signaling pathway. To test this hypothesis, we infected PD-1KO HCT mice, which were administered either HK *Lj* or PBS, and then monitored weight loss, survival, and the extent of lung fibrosis. WT HCT mice began to experience weight loss after 7 dpi with MHV-68, and the intranasal administration of HK *Lj* in WT HCT mice trended toward mitigating this MHV-68–induced weight loss, with effects becoming noticeable starting at 11 dpi (Fig. 7A). Compared to WT HCT mice, PD-1KO HCT mice experienced more pronounced weight loss starting at as early as 3 dpi with MHV-68 and succumbed to the infection by 12 dpi (Fig. 7, A and B). These outcomes are consistent with the protective role of PD-1 in preventing lethal immunopathology during acute viral infections (*45*). HK *Lj* administration attenuated both weight loss and mortality in the infected PD-1KO HCT mice (Fig. 7, A and B), suggesting that HK *Lj* generates some of the protective effects independent of PD-1. However, HK *Lj* failed to mitigate lung fibrosis as was observed in WT HCT counterparts (Fig. 7C). These results suggest that the PD-1 pathway plays an essential role in mediating the antifibrotic effect of HK *Lj* administration.

We extended our investigation into the role of PD-L1/PD-1 signaling in pulmonary fibrosis using an anti–PD-1–neutralizing antibody that has been shown to be effective in promoting antitumor immunity. The intranasal administration of HK *Lj* ameliorated MHV-68–induced weight loss in HCT mice; however, PD-1 neutralization in HK *Lj*–treated HCT mice abolished this protective effect (Fig. 7D). In addition, PD-1 neutralization was associated with increased mortality (Fig. 7E). Consistent with our findings in PD-1KO HCT mice, PD-1 neutralization in WT HCT mice inhibited effects of HK *Lj* administration on pulmonary fibrosis assessed by measuring lung collagen content (Fig. 7F) and evaluating histological evidence of fibrosis (Fig. 7, G and H). Both models demonstrate that PD-1 deficiency in HCT mice leads to increased disease severity following MHV-68 infection. Although HK *Lj* administration offers some protective effects, it is insufficient to reduce lung fibrosis in these PD-1–deficient mice. The mediators underlying HK *Lj*’s PD-1–independent effects remain unidentified. HK *Lj* was unable to decrease TGF-β and IL-17A levels in PD-1KO HCT mice at 7 dpi (Fig. 6, F and I), likely accounting for the persistent fibrosis observed. Together, these findings indicate that the PD-L1/PD-1 signaling axis in T_H_17 cells and macrophages is essential for the antifibrotic effects of HK *Lj* in HCT mice following viral infection.

### DCs recognize HK *L. johnsonii* through TLR2- and TLR9-MyD88 signaling

**Fig. 8.**
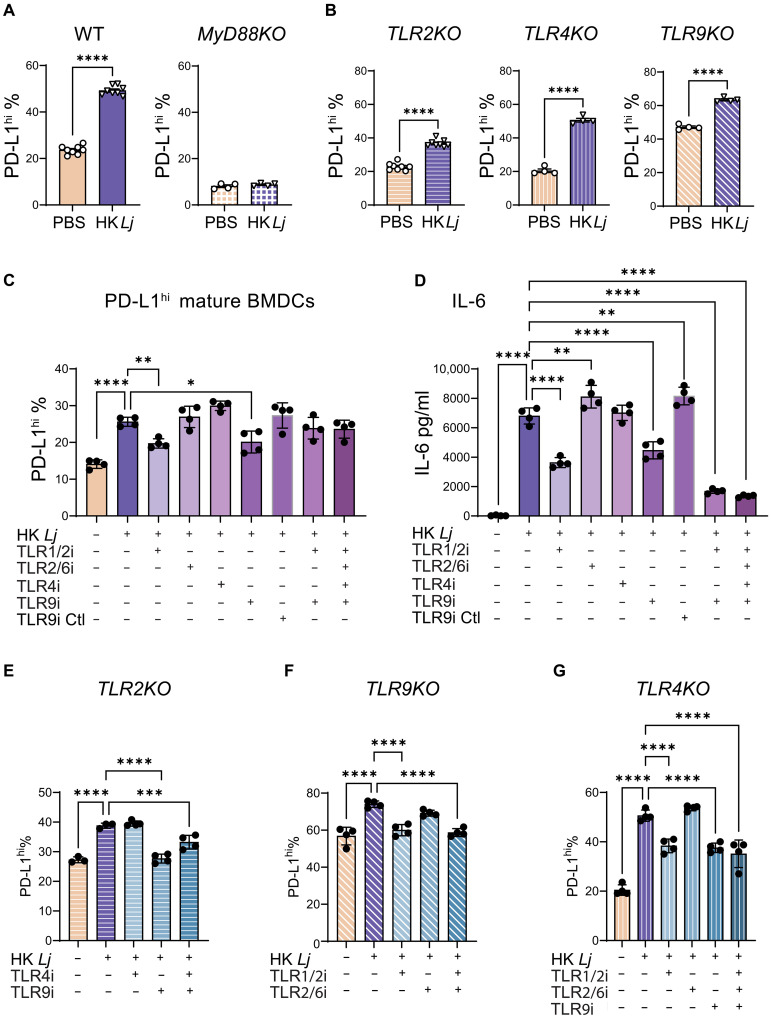
TLR4 and TLR9 signaling in HK *Lj* recognition and PD-L1 expression up-regulation on mature BMDCs. WT and mutant BMDCs were pretreated with DMSO, TLR1/2 inhibitor CU-CPT22 TLR2/6 inhibitor GIT27, TLR4 inhibitor C34, TLR9 antagonist ODN 2088, control oligos, or a combination of these inhibitors for 4 hours, followed by supplementation with HK *Lj* at a cell-to-bacteria ratio of 1:2 for 18 hours. (**A**) Frequency of PD-L1^hi^ mature WT and *MyD88KO* BMDCs with or without HK *Lj* treatment, as determined by flow cytometry analysis. (**B**) Frequency of PD-L1^hi^ mature *TLRKO* BMDCs with or without HK *Lj* treatment. (**C**) Frequency of PD-L1^hi^ mature WT BMDCs treated with HK *Lj* and TLR inhibitors. (**D**) Concentration of IL-6 in BMDC culture media after treatment with HK *Lj* and TLR inhibitor, determined by ELISA. (**E**) Frequency of PD-L1^hi^ mature *TLR2KO* BMDCs treated with HK *Lj*, TLR4, and TLR9 inhibitors. (**F**) Frequency of PD-L1^hi^ mature *TLR9KO* BMDCs after treatment with HK *Lj* and TLR1/2 and/or TLR2/6 inhibitors. (**G**) Frequency of PD-L1^hi^ mature *TLR4KO* BMDCs after treatments with HK *Lj* and TLR1/2, TLR2/6, and/or TLR9 inhibitors. Data are presented as means ± SEM. Statistical significance is denoted by *P < 0.05, **P < 0.01, ***P < 0.001, and ****P < 0.0001, as determined by one-way ANOVA with Tukey’s multiple comparisons test, comparing the mean of each group with the mean of the group treated with HK *Lj*. Results are representative of two independent experiments.

We next aimed to elucidate the mechanism by which DCs recognize HK *Lj* and up-regulate PD-L1 expression on their surface. The HK *Lj* supplement likely contains cell wall debris and cytoplasmic residues, including lipoteichoic acid, teichoic acid, peptidoglycan, and DNA fragments. These microbial components are recognized by various pattern recognition receptors, such as TLRs, Nod-like receptors (NLRs), and C-type lectin receptors (CLRs). MyD88 is an adaptor required for signaling by most TLRs except TLR3, but not by NLRs or CLRs. We used BMDCs generated from *MyD88KO* mice to determine the type of receptors that played a role in recognizing HK *Lj*. HK *Lj* treatment did not affect the survival of WT or *MyD88KO* BMDC (fig. S14A). HK *Lj* treatment of WT BMDCs significantly increased the frequency of mature DCs, but HK *Lj*–treated *MyD88KO* BMDCs remained at a low frequency of mature DCs (fig. S14B). Within the mature DCs, PD-L1^hi^ cell frequency increased considerably in WT but not in MyD88KO BMDCs (Fig. 8A). These findings indicate that TLRs, not NLRs or CLRs, are involved in the recognition and up-regulation of PD-L1 on DCs in response to HK *Lj*.

Among TLRs, TLR2 (*46*, *47*), TLR4 (*48*), and TLR9 (*49*, *50*) are known to play a role in lactobacilli recognition. To assess their importance in detecting HK *Lj* and regulating PD-L1 expression, we generated BMDCs from mice deficient in each of these receptors. TLRKO BMDC viability was unaffected by HK *Lj* treatment (fig. S14C), and all TLR-deficient BMDCs showed HK *Lj*–induced DC maturation (fig. S14D). The mature DC populations in each TLR mutant responded to HK *Lj* by increasing their PD-L1^hi^ populations (Fig. 8B). Hence, although TLR-MyD88 signaling is required for BMDCs to recognize HK *Lj* and up-regulate PD-L1, the deficiency of any single TLR does not prevent this response. This suggests that multiple TLR pathways may be involved in the recognition of HK *Lj* by BMDCs and that these signals may compensate for one another.

To further assess the importance of each TLR, we treated the same batch of WT BMDCs with various TLR inhibitors, independently and in combinations. TLR2 forms heterodimers with either TLR1 or TLR6 to transduce signals (*51*), with TLR1/2 signaling inhibited by CU-CPT22 (*52*) and TLR2/6 signaling by GIT27 (*53*). TLR4 and TLR9 signaling are inhibited by C34 (*54*) and oligodeoxynucleotide (ODN) 2088 (*55*), respectively. Except for TLR1/2 inhibitor CU-CPT22, which slightly decreased BMDC survival, other inhibitors showed no significant impact (fig. S15A). TLR1/2 or TLR9 inhibition resulted in reduced DC maturation upon HK *Lj* stimulation, implicating both receptors in HK *Lj* recognition (fig. S15B). Both inhibitors suppressed the HK *Lj*–induced increase in the PD-L1^hi^ population (Fig. 8C), but neither individual nor combined inhibition of TLR1/2 and TLR9 completely suppressed the increase in PD-L1^hi^ cells (Fig. 8C). Consistent with reports that TLR1/2 and TLR9 inhibition reduces IL-6 and IL-10 production in immune cells (*55*, *56*), we found that TLR1/2 or TLR9 inhibition alone suppressed IL-6 production induced by HK *Lj*, and simultaneous TLR1/2 and TLR9 inhibition more effectively minimized IL-6 production than did individual inhibition (Fig. 8D). Similar effects of TLR1/2 and/or TLR9 inhibitions were found on IL-10 production (fig. S15C).

To minimize potential interactions between inhibitors, TLR2KO BMDCs were treated with either the TLR4 inhibitor C34 or the TLR9 antagonist ODN 2088, and TLR9KO BMDCs were treated with TLR2 inhibitors CU-CPT22 or GIT27. Both TLR9-deficient BMDCs (TLR9 inhibition or knockout) showed reduced DC maturation in response to HK *Lj* (fig. S15, D and E), while CU-CPT22 slightly reduced the viability and DC maturation of TLR9KO or TLR4 BMDCs (fig. S15, E and F). TLR9 inhibition completely prevented the increase in the PD-L1^hi^ population in TLR2KO BMDCs post-HK *Lj* stimulation (Fig. 8E), and TLR2 inhibition entirely blocked the PD-L1^hi^ increase in TLR9KO BMDCs (Fig. 8F). These results demonstrate that in the absence of both TLR1/2 and TLR9 signaling, HK *Lj* cannot up-regulate PD-L1 expression on DCs. In TLR4KO BMDCs, similar to WT BMDCs, the PD-L1^hi^ population was partially inhibited by either CU-CPT22 or ODN 2088, and combining these inhibitors did not enhance suppression (Fig. 8G). This suggests a potential interaction between the effects of CU-CPT22 and ODN 2088. Because both TLR1/2 and TLR9 contribute to PD-L1 up-regulation on BMDCs, we tested whether stimulation with their respective agonists would be sufficient to induce PD-L1 expression. We found that stimulation of BMDCs with either a TLR2 agonist (Pam3CSK4) or a TLR9 agonist (ODN 1826) alone was insufficient to up-regulate PD-L1 (fig. S16). Together, these findings indicate that while TLR1/2 and TLR9 pathways are important for DCs to recognize HK *Lj* and up-regulate PD-L1, they are not sufficient on their own, suggesting that additional signals from HK *Lj* are required for PD-L1 up-regulation on DCs.

## DISCUSSION

We have demonstrated that intranasal administration of low-dose nonviable *L. johnsonii* XZ17, a bacterium commonly present in healthy mouse lungs but diminished after HCT, alleviates pneumonitis and pulmonary fibrosis in HCT mice infected with MHV-68. This protective effect is mediated by the up-regulation of PD-L1 on DCs in virally infected HCT mice, leading to dampened IL-17A production in T_H_17 cells. Our results show that DCs recognize *L. johnsonii* and up-regulate PD-L1 expression through the TLR1/2- and TLR9-MyD88 pathways. Moreover, the administration of HK *L. johnsonii* reduces both pro-inflammatory and profibrotic cytokine production by lung macrophages, effects that are also dependent on PD-1 signaling.

T_H_17 cells and macrophages are pivotal mediators in the pathogenesis of pulmonary fibrosis, primarily through their respective cytokines. IL-17, produced by T_H_17 cells, drives chronic inflammation and fibroblast activation, contributing to tissue remodeling and fibrotic progression (*16*, *57*–*59*). While most mechanistic insights into T_H_17 involvement in IPS have come from animal models, emerging clinical evidence links increased T_H_17 frequencies and IL-17A levels with lung injury in human IPS after HCT (*18*). These findings emphasize the clinical relevance of strategies that modulate T_H_17-driven pathways in IPS. Similarly, macrophage-derived TGF-β is a major profibrogenic cytokine, directly stimulating extracellular matrix deposition and myofibroblast differentiation (*34*, *60*, *61*). The interplay between IL-17 and TGF-β amplifies immune responses and perpetuates the fibrotic cascade, highlighting the importance of targeting these cells and cytokines for fibrosis prevention and treatment (*62*, *63*).

The regulation of PD-L1 on DCs is complex. While interferon-γ (IFN-γ) is a major inducer of PD-L1 via the Janus kinase–signal transducers and activators of transcription and interferon regulatory factor (IRF) pathways (*64*), TLR signaling also activates nuclear factor κB and IRF pathways, which may directly or indirectly influence PD-L1 expression. The modulation of PD-L1 expression through TLR2 and TLR9 has been documented (*65*–*68*), but the underpinnings remain incompletely understood. Our study shows that TLR1/2 and TLR9 stimulation by HK *Lj* increases IL-6 and IL-10 production. While IL-6 generally promotes inflammation and fibrosis, IL-10 is often anti-inflammatory and antifibrotic; notably, both up-regulate PD-L1 (*69*, *70*), suggesting potential paracrine or autocrine feedback mechanisms for PD-L1 regulation. Since IL-6 is essential for the differentiation of T_H_17 cells, it is plausible that, while IL-6 directly promotes T_H_17 cell polarization, it might also indirectly suppress T_H_17 cell activity through up-regulation of PD-L1 on DCs (*71*). The overall impact of IL-6 released from DCs on T_H_17 polarization and functional regulation in this HK *Lj*–administered HCT mouse model remains to be clarified. Meanwhile, our in vivo data demonstrate that HK *Lj* suppresses IL-6, other inflammatory cytokines, and profibrogenic TGF-β in macrophages, the main lung source of IL-6, in a PD-1–dependent manner. Therefore, outcomes in pathology and remodeling are ultimately shaped by multiple cell types, and the roles of each may vary depending on the lung injury’s etiology.

Our work also highlights the critical importance of TLR1/2 and TLR9 in recognizing HK *Lj* and inducing PD-L1 expression on DCs. Notably, TLR1/2 recognizes triacylated lipopeptides (*72*), more commonly found in Gram-negative bacteria. As a Gram-positive organism, *L. johnsonii* lacks the apolipoprotein *N*-acyltransferase required for synthesizing triacylated lipoproteins, suggesting that alternative ligands or altered protein structures following heat inactivation contribute to TLR1/2 activation. Previous studies have shown that *L. johnsonii* can engage TLR1/2 and alleviate colitis in mice (*47*). TLR9 recognizes unmethylated CpG motifs in bacterial DNA (*73*), and heat treatment of HK *Lj* may enhance DNA exposure, promoting TLR9 activation. Studies in *L. johnsonii* N6.2 have found that extracellular vesicles containing DNA stimulate TLR9 signaling (*49*, *74*). Neither the synthetic TLR1/2 agonist Pam3CSK4 nor the TLR9 agonist ODN 1826 alone induced PD-L1 up-regulation on BMDCs. Similarly, HK Gram-positive *L. murinis* or MRSA bacteria did not reduce fibrosis in HCT mice. Together, our data suggest that HK *Lj*’s ability to induce PD-L1 expression and protect against lung fibrosis depends on unique cellular components that warrant further identification.

*L. johnsonii* is frequently detected in healthy mouse lungs but is likely a transient visitor rather than a permanent resident. The lung microbiome is a dynamically regulated, low-biomass community, often reflecting upper respiratory tract composition (*75*, *76*). Bacterial entry occurs mainly via microaspiration, not contiguous mucosal spread (*76*, *77*). The harsh pulmonary environment limits bacterial survival, illustrated by a two-log drop in lung bacterial count just 1 day after live *L. johnsonii* XZ17 instillation in HCT mice. This rapid clearance supports the use of nonviable bacteria as therapeutics. Coprophagy in mice allows gut-to-lung microbial transfer, consistent with our previous finding of a parallel loss of *Lactobacillus* species in both lung and gut after HCT (*8*). Similarly, lactobacilli are abundant in the human oral cavity (*78*), and oral-lung migration can influence pulmonary fibrosis risk (*79*).

Nonviable microbial products or postbiotics are increasingly recognized for their safety and therapeutic potential (*80*). Live lactobacilli are considered safe for healthy individuals but may pose a risk of opportunistic infection in immunocompromised hosts, such as HCT recipients (*81*). In addition, many *L. johnsonii* strains harbor antibiotic resistance genes (*82*). Clinical studies demonstrate the safety and benefit of HK *L. johnsonii* in humans (*83*). Our findings support that nonviable HK *Lj* is safe and effective in attenuating pulmonary fibrosis in an immunocompromised host, providing a rationale for developing postbiotic therapies for HCT complications.

The immunosuppressive mechanism of *L. johnsonii* may have context-specific consequences. While augmenting PD-L1/PD-1 signaling may suppress inflammation and fibrosis, it could also hinder antitumor immunity. For example, a large retrospective cohort of patients with melanoma showed that probiotics could negate the benefits of dietary fiber on immune checkpoint blockade efficacy (*84*), and mice treated with *Lactobacillus rhamnosus* GG or *Bifidobacterium longum* had reduced responses to anti–PD-L1 therapy (*84*). In acute graft versus host disease (aGVHD), data remain mixed: *L. rhamnosus* GG had no effect in a small cohort (*85*), whereas a multistrain probiotic and prebiotic regimen was beneficial in a recent study (*86*). Furthermore, in cancer models, live but not HK *L. johnsonii* enhanced PD-1 cancer therapy response via indole-3-propionic acid production (*87*). These findings underscore the complexity and context specificity of immune modulation by lactobacilli.

The role of PD-L1/PD-1 signaling in pulmonary fibrosis may depend on the specific disease context. IPF, bleomycin-induced fibrosis, and virus-induced post-HCT fibrosis each have distinct mechanisms (*88*). Elevated PD-L1 and PD-1 levels have been observed in patients with IPF and animal models (*89*, *90*), which differs from our findings, where HK *Lj*–induced PD-L1/PD-1 signaling reduced fibrosis after MHV-68 infection and HCT. This highlights the divergent roles for immune pathways in different fibrosis contexts. For example, CCL2/CCR2 signaling drives fibrotic pathology in bleomycin models (*32*) but actually helps control severity in herpesvirus-induced fibrosis post-HCT (*91*).

In summary, this study provides a promising and potentially safe therapeutic approach using postbiotics for the respiratory mitigation of severe pulmonary complications in HCT recipients. Our findings elucidate a mechanism by which heat-inactivated *L. johnsonii* suppresses T_H_17 cell activity via TLR1/2 and TLR9 recognition and increased PD-L1 expression on DCs and reduces pro-inflammatory and profibrotic cytokine production by lung macrophages. This work lays the foundation for future studies, including monitoring the oral microbiome as a predictor for post-HCT lung complications, developing targeted lung microbiome interventions, identifying the active components of *L. johnsonii*–mediating DC responses, and uncovering mechanisms of DC-postbiotic interaction.

## MATERIALS AND METHODS

### Mice

C57BL/6J (B6, JAX000664), B6.Cg-Pdcd1^tm1.15hr^/J (PD-1KO, JAX28276), B6.129P2(SJL)-Myd88^tm1.1Defr^/J (MyD88KO, JAX009088), B6.129-Tlr2^tm1Kir^/J (TLR2KO, JAX004650), B6(Cg)-Tlr4^tm1.2Karp^/J (TLR4KO, JAX029015), and B6.C-Tg(CMV-cre)1Cgn/J (CMV-Cre, JAX006054) mice were purchased from the Jackson Laboratory (Bar Harbor, ME), and TLR9^fl/fl^ mice on the C57B/6 background were provided by G. Sen (Cleveland Clinic). CMV-Cre-TLR9^fl/fl^ (TLR9KO) mice, which have TLR9 deleted in all tissues, were generated crossing CMV-Cre and TLR9^fl/fl^ mice. The mice were housed in a specific pathogen–free facility at University of Michigan. Upon reaching the predetermined end points, mice were euthanized using CO_2_ (Cryogenic Gases, Detroit, MI), and organs were harvested for subsequent cell preparation or assay procedures. All procedures involving mice were approved by the University of Michigan’s Institutional Animal Care and Use Committee, as per the protocol numbers PRO00010437 and PRO00011303.

### Syn-HCT and MHV-68 infection

Recipient B6 mice (7 to 9 weeks old) received a total body irradiation of 13 grays by a ^137^Cs irradiator, administered in two split doses 3 hours apart. Subsequently, 5 × 10^6^ whole bone marrow cells, harvested from B6 or PD-1KO mice, were infused via tail vein injection. At 5 weeks post-HCT, the mice were inoculated with MHV-68 [VR-1465, American Type Culture Collection (ATCC)]. To do that, both non-HCT and HCT mice were anesthetized using a mixture of ketamine and xylazine, followed by the intranasal administration of 5 × 10^4^ PFU MHV-68.

### Isolation and identification of *L. johnsonii*

A lung from a healthy naïve adult B6 mouse was homogenized in 1 ml of PBS under aseptic conditions. Lung homogenate was then streaked onto *Lactobacillus*-selective de MRS agar (BD Biosciences) for 24 hours at 37°C under an aerobic environment supplemented with CO_2_. Pure cultures of eight colonies were established by restreaking and subsequently maintained as frozen stocks. Bacterial identification was performed through polymerase chain reaction (PCR) amplification of a segment of the 16*S* rRNA gene using the degenerate primers D88 and E94 (*23*). Sequencing analysis confirmed that all isolates were genetically identical, displaying 100% sequence homology with the 16*S* rRNA gene sequence of *L. johnsonii* (*24*). A single isolate, designated *L. johnsonii* XZ17, was selected for full genomic sequencing (*20*). The genome of XZ17 was deposited under GenBank accession number CP151183.1.

### Phylogenetic analysis on *L. johnsonii* genomes

Phylogenomic analysis was carried out on complete genome sequences of 17 *L. johnsonii* strains sourced from a variety of vertebrate hosts including humans, rodents, swine, and poultry. These strains are BS15, ATCC 33200, IDCC9203, NCC 533, CH32, FI9785, UMNLJ21, UMNLJ22, ZLJ010, PF01, DPC 6026, Byun-jo-01, MR1, MT4, PC38, and NCK2677. The type strain ATCC 33323 of *Lactobacillus gasseri*, the species most closely related to *L. johnsonii*, was used as the out-group external reference. ANI analyses of whole genomes were conducted using fastANI (*92*). The resulting pairwise ANI scores were used to create a Euclidean distance matrix, from which a phylogenetic tree was constructed using hierarchical clustering in R. The final phylogenetic tree was visualized using Phylo.io (*93*), enabling the elucidation of the genetic relationships among the strains.

### Preparation and administration of *L. johnsonii*, *L. murinus*, or MRSA

For the preparation of *L. johnsonii* XZ17 stocks, 100 ml of MRS broth was inoculated with the bacterium from a frozen glycerol stock and cultured statically overnight at 37°C. The stationary-phase cultures were pelleted by centrifugation at 1500*g* for 5 min at 4°C and subsequently resuspended in 50 ml of a 1:1 (v/v) solution of MRS broth and 50% glycerol. Aliquots of 500 μl of the bacterial suspension were stored at −80°C. The viable cell counts of the prepared frozen glycerol stocks were determined as 1 × 10^9^ CFU per vial through serial dilution.

To administer live *L. johnsonii* XZ17, stocks were rapidly thawed at 37°C, pelleted by centrifugation at 1500*g* for 2 min, and washed with PBS to eliminate excess glycerol. The washed cells were resuspended in sterile PBS to a final concentration of 1 × 10^4^ CFU/μl for intranasal delivery (50 μl per mouse) or to a concentration of 4 × 10^5^ CFU/μl for oral gavage (100 μl per mouse). Before intranasal administration, mice were sedated with isoflurane. The viability of *L. johnsonii* was confirmed by plating the remaining suspension on MRS agar throughout the experiment.

To generate HK *L. johnsonii* XZ17 stocks, a vial of the frozen stock was quickly thawed at 37°C, washed with sterile PBS, resuspended in 1 ml of PBS, and then subjected to a 70°C water bath for 15 min to inactivate the bacteria. The HK cells were pelleted, resuspended in sterile PBS to a final concentration equivalent to 1 × 10^6^ CFU/μl before HK, and aliquoted into 15-μl vials, which were then stored at −80°C. The inactive status of the HK *L. johnsonii* stocks was confirmed by the absence of growth after 2 days on MRS agar. For intranasal administration of HK *L. johnsonii*, 1.5 ml of PBS was added to a stock vial to prepare the inoculum, and 50 μl per mouse (equivalent to 5 × 10^5^ CFU before HK) was administered intranasally.

*L. murinus* (*94*) was purchased from ATCC (35020). HK *L. murinus* was prepared using the same protocol as for HK *L. johnsonii* and administered intranasally at a dose equivalent to 5 × 10^5^ CFU before HK per mouse. MRSA strain USA300-0114 was obtained from the Network of Antimicrobial Resistance in *S. aureus*. To prepare bacterial cultures, MRSA was grown overnight at 37°C with constant shaking in Nutrient Broth (BD Difco, Franklin Lakes, NJ). CFU counts were estimated by optical density using a standard curve. Bacterial cells were resuspended in sterile PBS at a concentration of 1 × 10^6^ CFU/μl and HK at 85°C for 15 min. The inactivation of HK MRSA was confirmed by the absence of growth after 2 days on Nutrient agar. Mice received an intranasal dose equivalent to 1 × 10^6^ CFU before HK per mouse.

### Plaque assay

The quantification of lytic MHV-68 virus from mouse right lungs was performed by plaque assay as previously described (*95*). Briefly, right lungs were harvested at 3 or 7 dpi and then homogenized in 1 ml of Dulbecco’s modified Eagle’s medium containing 10% fetal calf serum and complete protease inhibitor (Roche, MilliporeSigma). Homogenate supernatants were serially diluted and applied in duplicate to confluent monolayers of 3T12 cells (ATCC, CCL-164). Plaques were enumerated after 7 days of incubation.

### PD-1 blockade in HCT mice

Mice that underwent the HCT procedure and received HK *L. johnsonii* supplementation were intraperitoneally administered 100 μg of rat anti–PD-1 monoclonal neutralizing antibodies (clone 29F.1A12, Bio X Cell). The administration was carried out at intervals of 2 to 3 days, beginning at 2 dpi with MHV-68 and continuing through 7 or 21 dpi.

### RNA extraction and qPCR analysis

RNA was isolated from lung tissues or specific cell types using TRIzol reagent (Invitrogen, Thermo Fisher Scientific) according to the instructions provided by the manufacturer. The concentration and purity of the extracted RNA were evaluated using a NanoDrop Lite Spectrophotometer (Thermo Fisher Scientific). Gene expression levels were quantified by reverse transcription quantitative PCR (RT-qPCR) using the Luna Universal Probe One-Step RT-qPCR Kit (New England Biolabs) and being conducted on a QuantStudio 3 Real-Time PCR System (Applied Biosystems). Detailed information on the primer and probe sets used in this study can be found in table S1.

### Hydroxyproline assay

The hydroxyproline content in lung tissue was quantified using an assay modified from previously described methods (*32*). In summary, individual lung lobes were isolated and homogenized in 1 ml of PBS and subsequently hydrolyzed through the addition of 1 ml of 12 N HCl (Sigma-Aldrich), followed by incubation at 120°C for 18 hours. The assay was carried out in a 96-well plate, where 5 μl of each hydrolysate sample and a series of standard concentrations of cis-4-hydroxy-l-proline (MilliporeSigma) were incubated with a chloramine T (MilliporeSigma) solution for 20 min. This was followed by the addition of Ehrlich’s reagent (MilliporeSigma) and a further incubation at 65°C for 15 to 20 min to induce color development. The absorbance of each sample was then measured at 550 nm using a BioTek Synergy H1 Plate Reader (Agilent Technologies).

### Lung section and fibrosis scores

Whole lungs were prepared for fibrosis assessment by first being perfused and inflated before fixation in 10% buffered formalin. Following fixation, the tissues were subjected to dehydration through an ethanol series and embedded in paraffin. Tissue sections of 3-μm thickness were then sectioned, placed on glass slides, and stained using the Masson’s trichrome technique. Photomicrographs of the stained sections were captured using a Nikon Eclipse E400 microscope equipped with an Olympus EP50 camera. For each stained section, five fields were imaged at ×100 magnification. The severity of fibrosis within these images was quantitatively evaluated using the Modified Ashcroft scoring method (*96*).

### Lung cell preparation and flow cytometry analysis

To prepare single-cell suspensions from lung tissue for flow cytometry, whole lung collagenase digestion was performed as previously described (*16*). Briefly, mice were euthanized with CO_2_, followed by perfusion of the lungs via the right ventricle with 5 ml of PBS. For intravascular immune cell staining (*97*), 2 μg of anti-mouse CD45.2 antibody (104, BioLegend) diluted in 200 μl of PBS was injected into the tail vein of each mouse 3 min before euthanasia. The lungs were then excised, and lung lobes were finely minced and incubated in 15 ml of complete media containing collagenase (1 mg/ml; Roche) and deoxyribonuclease I (17 U/ml; Sigma-Aldrich) at 37°C for 30 min. Tissue homogenization was achieved by repeatedly drawing the digested mixture through the bore of a 10-ml syringe, followed by filtration through a 100-μm mesh to obtain a suspension of single cells. Red blood cells were lysed in ammonium chloride-potassium buffer. Leukocytes were further enriched by gradient centrifugation in 20% Percoll in culture media at 2000*g* for 20 min. For intracellular staining, cell suspensions were stimulated with PMA (0.05 μg/ml) and ionomycin (0.75 μg/ml; both from Sigma-Aldrich) in the presence of the GolgiStop protein transport inhibitor (BD Pharmingen) for 4 hours. Before staining, 1 × 10^6^ cells were blocked using anti-CD16/CD32 antibodies (Fc block; BD Pharmingen) and then labeled with a panel of fluorochrome-conjugated antibodies including CD45 (30-F11, BD Pharmingen), CD11c (N418, eBioscience), CD11b (M1/70, BD Horizon), I-A^b^ (AF6–120.1, BD Horizon), Siglec F (E50-2440, BD Horizon), CD103 (M290, BD Bioscience), Ly6G (1A8, BioLegend), CD90.2 (30-H12, BioLegend), PD-L1 (10F.9G2, BioLegend), Lg6C (HK1.4, BioLegend), Ep-CAM (G8.8, BioLegend), CD64 (X54-5/7.1, BioLegend), CD26 (H194-112, BD Bioscience), FcεRIα (Mar-1, BioLegend), Tim-3 (B8.2C12, BioLegend), CD24 (M1/69, BioLegend), T cell receptor γδ (GL3, BD Horizon), CD4 (GK1.5, BioLegend), PD-1 (J43, BD Horizon), CD3 (17A2, BD Pharmingen), CD8a (53-6.7, BioLegend), and CCR6 (29-2 L17, BioLegend). After cell surface staining, the cells were then fixed and permeabilized with Foxp3 staining buffer set (eBioscience) and stained for intracellular tumor necrosis factor–α (MP6-XT22, BioLegend), RORγT (Q31-378, BD Pharmingen), FoxP3 (FJK-16 s, eBioscience), IFN-γ (XMG1.2, BD Pharmingen), and IL-17a (TC11-18H10.1, BioLegend). Flow cytometry was conducted on a Cytek Aurora analyzer (Cytek Biosciences) or a BD LSRFortessa Cell Analyzer. Data were analyzed by FlowJo software version 10.9.0 (FlowJo, LLC).

### Macrophage and CD4 T cell isolation

Macrophages were isolated from lung single-cell suspensions using microbeads conjugated to anti-F4/80 antibodies according to the manufacturer’s protocol (Miltenyi Biotec). CD4^+^ T cells were negatively enriched from the lung single-cell suspensions using the MojoSort Mouse CD4 T Cell Isolation Kit (BioLegend) in alignment with the manufacturer’s guidelines.

### Generation of BMDCs

BMDCs were generated from hematopoietic stem cells harvested aseptically from the femurs of 6- to 8-week-old C57BL/6J mice. The collected bone marrow cells were resuspended in complete RPMI 1640 medium (Gibco) supplemented with recombinant murine GM-CSF (10 ng/ml; R&D Systems) to achieve a final concentration of 5 × 10^5^ cells/ml. One milliliter of the cell suspension was aliquoted into each well of a 24-well tissue culture plate (Fisherbrand), and the cells were cultured for 6 days to facilitate differentiation into DCs. BMDCs were harvested and cryopreserved in a solution of 90% fetal bovine serum and 10% dimethyl sulfoxide (DMSO) until needed.

### Generation of iT_H_17 cells

The in vitro differentiation of T_H_17 cells was conducted in accordance with a previously published protocol (*98*). Briefly, splenocytes were harvested by mechanical disruption of the spleens from C57BL/6J or PD-1KO mice, passing the tissue through a 100-μm nylon cell strainer with the aid of a 3-ml syringe plunger. Following splenocyte collection, naïve CD4^+^ T cells were isolated using a negative selection kit (STEMCELL Technologies). The purified naïve CD4^+^ T cells, plated at a density of 3 × 10^5^ cells per 200 μl per well in a 96-well plate, were then stimulated with plate-bound anti-CD3 (clone 17A2, Invitrogen) and anti-CD28 (clone 37.51, Invitrogen) antibodies. The differentiation into T_H_17 cells was supported by a cytokine milieu consisting of recombinant IL-6, TGF-β, and IL-23 (all from R&D Systems), in conjunction with neutralizing antibodies against mouse IFN-γ and IL-4 (also from R&D Systems). Culturing was performed in Iscove’s modified Dulbecco’s medium (Gibco) supplemented with the necessary growth factors. Cells were incubated for 3 days to promote the generation of iTH17 cells.

### Stimulation of BMDCs with HK *L. johnsonii* and coculture with iTH17 cells

Cryopreserved BMDCs were thawed and cultured in complete RPMI 1640 medium supplemented with recombinant murine GM-CSF at a density of 2 × 10^5^ cells per 200 μl per well in a 96-well plate at 37°C incubated for 2 days, before being treated with HK *L. johnsonii* at a 1:2 ratio (BMDCs:HK *L. johnsonii*) for 18 hours. In experiments investigating the role of TLRs in the recognition of HK *Lj*, WT and mutant BMDCs were pretreated with either DMSO, 10 μM TLR1/2 inhibitor CU-CPT22 (Tocris Bioscience), TLR2/6 inhibitor GIT27 (10 μg/ml; Tocris Bioscience), 10 μM TLR4 inhibitor C34 (Tocris Bioscience), 2.5 μM TLR9 antagonist ODN 2088, its control oligos (InvivoGen), or a combination of these inhibitors for 4 hours, followed by supplementation with HK *Lj* for 18 hours. In experiments determining the effects of TLR1/2 or TLR9 agonist on BMDCs, cells were treated with Pam3CSK4 (300 ng/ml, a TLR1/2 agonist, InvivoGen), or ODN 1826 (5 μM, a TLR9 agonist, InvivoGen) for 18 hours. Supernatants were collected for determining the production of IL-6 and IL-10 (DuoSet ELISA kits, R&D Systems) in accordance with the manufacturer’s directions. To evaluate the surface expression of PD-L1 on BMDCs, cells were stained with antibodies against CD11c (clone N418, eBioscience), CD11b (clone M1/70, BD Horizon), I-Ab (clone AF6–120.1, BD Horizon), CD64 (clone X54-5/7.1, BioLegend), and PD-L1 (clone 10F.9G2, BioLegend). In coculture experiments, the HK *Lj*–stimulated BMDCs were washed twice with PBS and then cocultured with iT_H_17 cells differentiated from either B6 or PD-1KO mice using a BMDC:iT_H_17 cell ratio of 1:5. After 24 hours of coculture, the concentration of IL-17A in the culture supernatant was quantified using the Mouse IL-17 DuoSet ELISA Kit (R&D Systems) in accordance with the manufacturer’s directions.

### Determination of bacterial viability in lung homogenate

The lung homogenate was centrifuged at 300*g* for 2 min to pellet cell debris. The supernatant, typically 10 μl in volume, was then combined with an equal volume of 2X LIVE/DEAD *Bac*Light bacterial viability staining solution (Molecular Probes, Invitrogen). The mixture was kept in the dark for 15 min to allow the stain to penetrate the bacteria. The stained bacterial suspension was examined with a Zeiss Axioplan 2 fluorescence microscope to differentiate between live and dead bacteria. Images were captured using an Olympus DP28 camera to document and analyze the proportions of viable bacteria.

### Statistical analysis

Statistical analyses were conducted using GraphPad Prism version 9.0 (GraphPad Software). For comparisons between two groups, significance was assessed using two-tailed Student’s *t* tests for data with a normal distribution, and two-tailed Mann-Whitney *U* tests were applied for data without a normal distribution. When comparing three or more groups, one-way analysis of variance (ANOVA) followed by Tukey’s multiple comparisons test was used to evaluate the significance between groups.
