# Supplementary material for: Targeted lung Lactobacillus johnsonii intervention alleviates virus-induced fibrosis post-HCT through PD-L1/PD-1 signaling
Source: Sci Adv. 2026 Jan 1;12(1):eadw4654. doi: 10.1126/sciadv.adw4654 (PMC13150718; doi:10.1126/sciadv.adw4654)
Supplement: Supplementary file 1 — Figs. S1 to S16 Table S1 [file sciadv.adw4654_sm.pdf]

Science Advances
AAAS

Supplementary Materials for

Targeted lung *Lactobacillus johnsonii* intervention alleviates virus-induced fibrosis post-HCT through PD-L1/PD-1 signaling

Joshua B. Perkins *et al.*

Corresponding author: Xiaofeng Zhou, xiazhou@med.umich.edu; Bethany B. Moore, bmoore@med.umich.edu

Sci. Adv. **12**, eadw4654 (2026)
DOI: 10.1126/sciadv.adw4654

This PDF file includes:

Figs. S1 to S16
Table S1

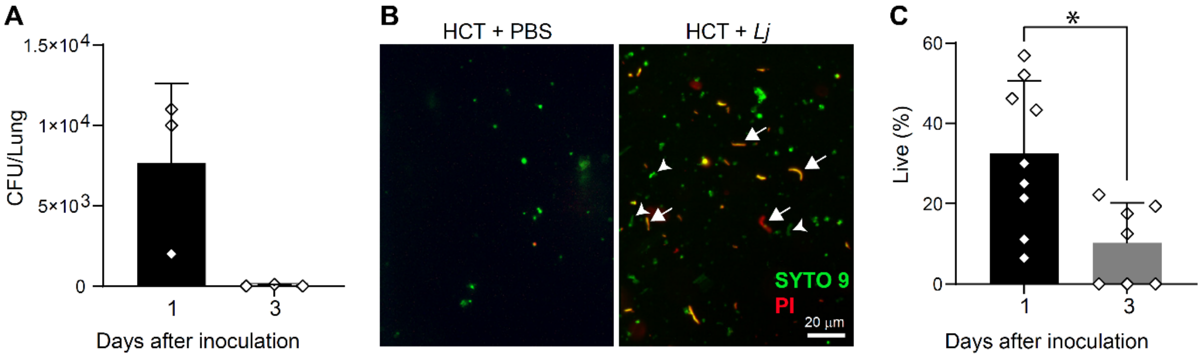

Fig. S1. Intranasally inoculated *L. johnsonii* XZ17 fails to establish colonization in the lungs of HCT mice.

(**A**) Colony-forming units (CFU) were assessed by culturing lung homogenates on MRS agar one or three days post-inoculation with 5×10^5 CFU of *L. johnsonii* XZ17. (**B**) Representative image showing live/dead staining of lung isolates from HCT mice three days after inoculation with *L. johnsonii* XZ17 (HCT+*Lj*) or vehicle (HCT+PBS). Arrowheads highlight live, rod-shaped bacteria stained green, while arrows indicate dead or dying bacteria stained red or yellow, respectively. (**C**) Percentage of live, rod-shaped bacteria in lung isolates from HCT mice one or three days after inoculation with *L. johnsonii* XZ17. Data are presented as mean ± SD. **P* <0.05, determined by unpaired two-tailed Student's *t*-tests. Results shown are representative of two independent experiments.

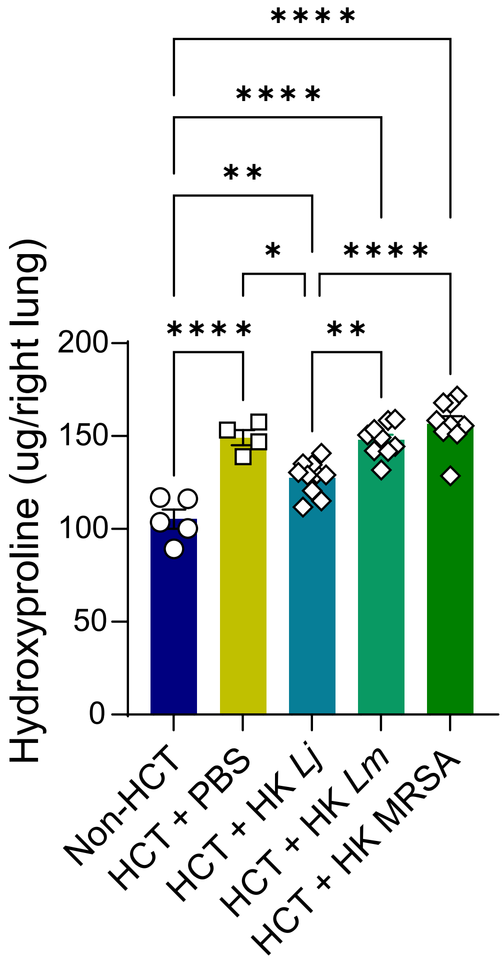

Fig. S2. The ability of HK *L. johnsonii* to attenuate lung fibrosis in MHV-68–infected HCT mice is unique to this bacterium.

Non-HCT C57BL/6J mice and HCT mice with mock, HK *Lj*, HK *Ligilactobacillus murinus* (*Lm*) or HK methicillin-resistant *Staphylococcus aureus* (MRSA) administration (n = 4~10 per group, 5 x10^5 erstwhile CFU of HK bacteria per mouse, every 2~3 days from -7 dpi to 19 or 20 dpi) were euthanized at 21 dpi with MHV-68, and their right lungs were collected for hydroxyproline assay. Data are presented as mean ± SEM. Statistical significance is indicated by *P <0.05; **P < 0.01; ****P < 0.0001, as determined by one-way ANOVA with Tukey's multiple-comparisons test.

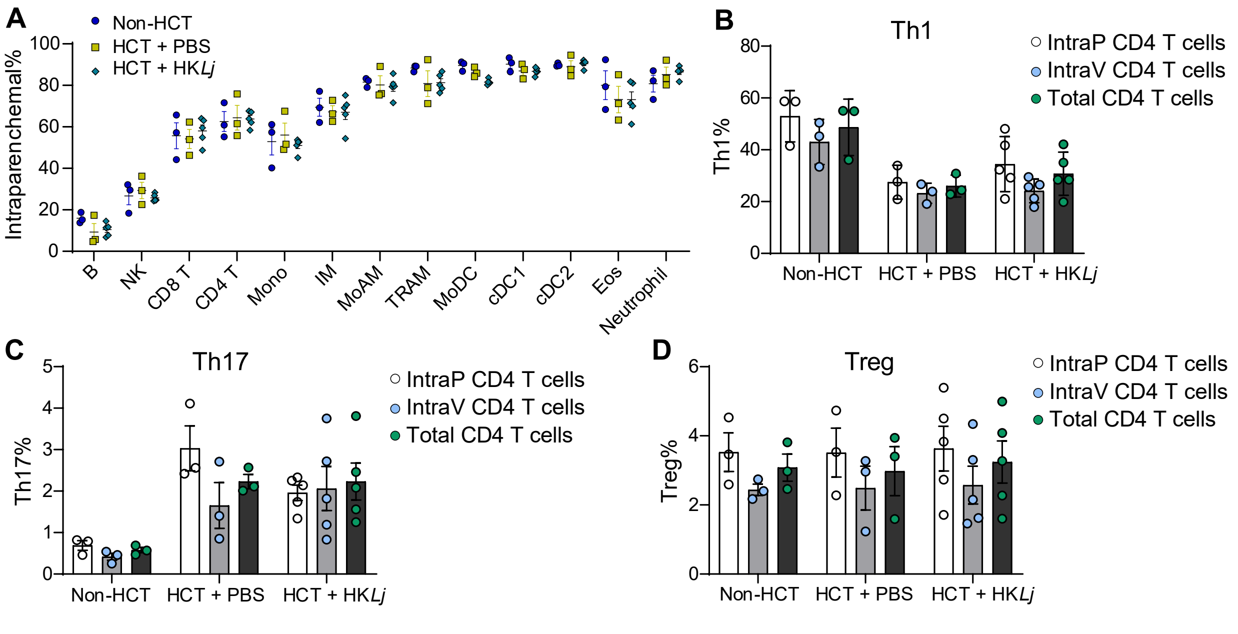

Fig. S3. Intraparenchymal and intravascular immune cells identified by intravenous anti-CD45.2 antibody labeling.

Non-HCT C57BL/6J mice and HCT mice with mock or HK *Lj* administration (n = 3~6 per group) were scheduled for euthanasia at 7 dpi. Two micrograms of anti-mouse CD45.2 antibody diluted in 200 µl PBS were injected into each mouse via the tail vein. Mice were euthanized three minutes after injection, and the lungs were perfused with approximately 10 ml PBS. Lungs were then collected and processed into single-cell suspensions for subsequent antibody staining and flow cytometry analysis. (**A**) Percentage of each immune cell type located within the intraparenchymal compartment. (**B–D**) Percentage of Th1 (**B**), Th17 (**C**), and Treg (**D**) cells among intraparenchymal (IntraP), intravascular (IntraV), or total CD4$^+$ T cell populations.

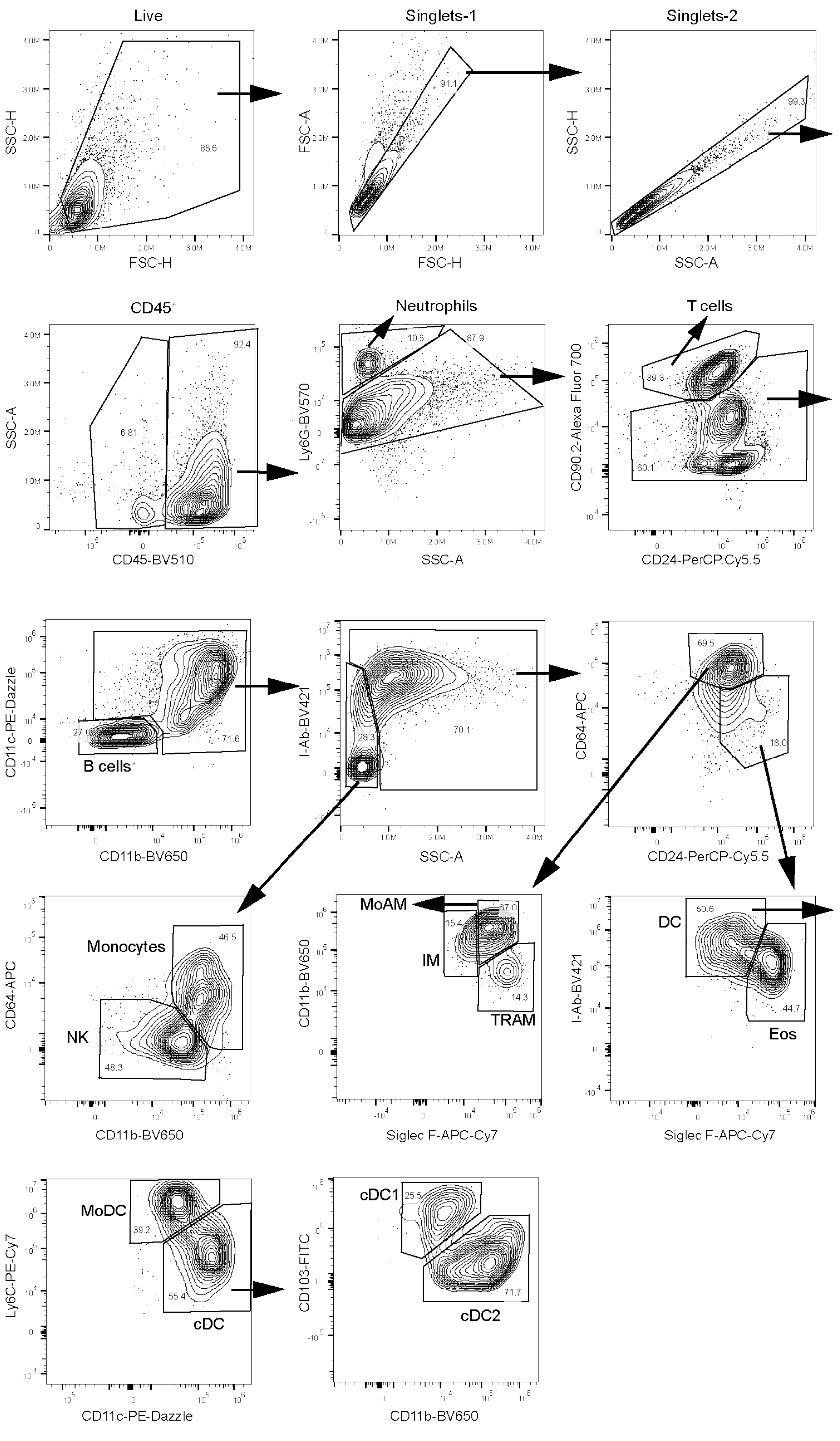

Fig. S4. Gating strategy lung immune cells.

Gating strategy used for FACS analysis of immune cell populations in the lungs 7 days post-infection with MHV-68.

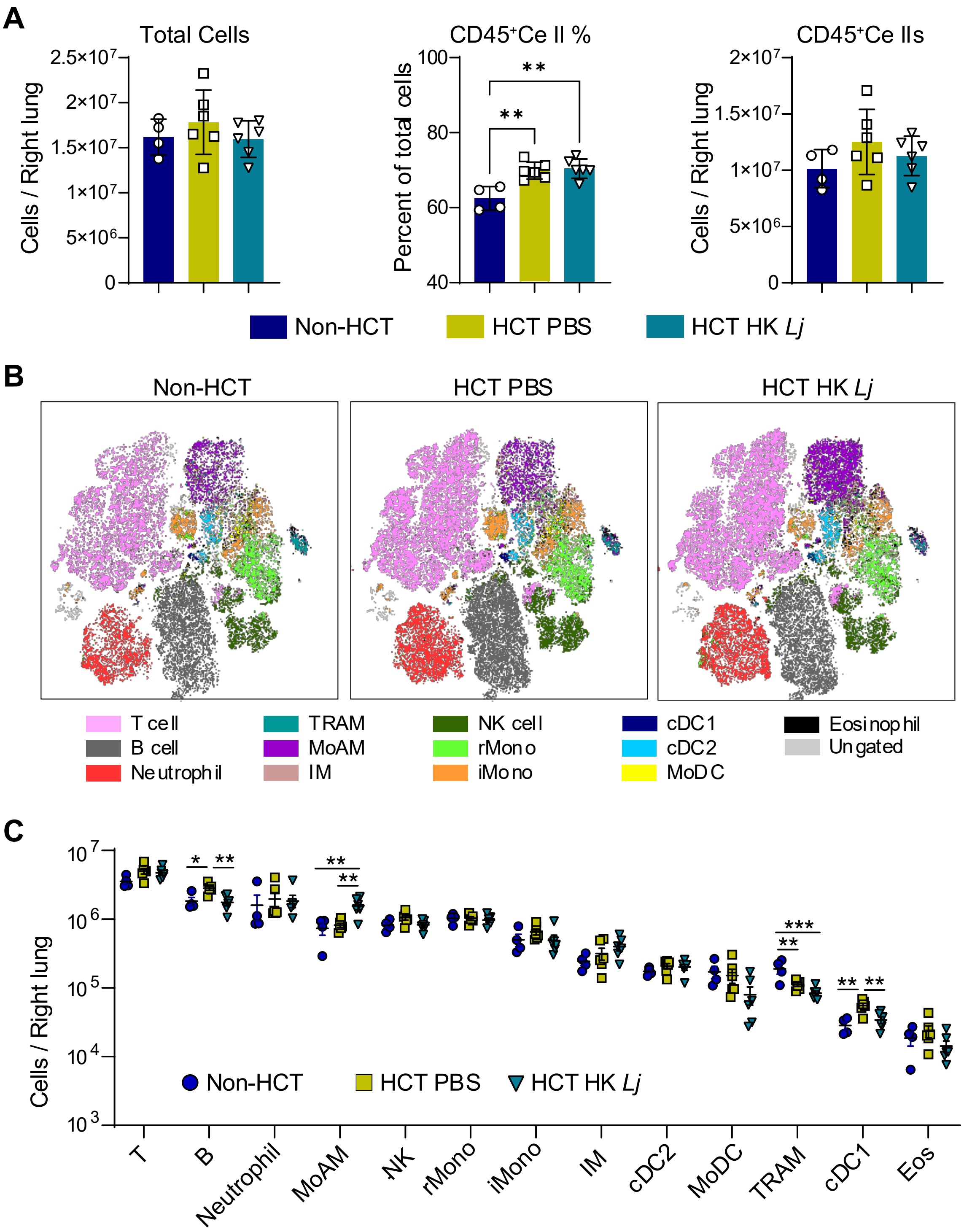

Fig. S5. Flow cytometry analyses of lung immune cells in lung tissue following MHV-68 infection at 7 dpi.

Non-HCT C57BL/6J mice (n = 4) and HCT mice with mock or HK *Lj* administration (n = 6 per group) were euthanized at 7 dpi, and their lung single-cell suspensions were prepared for flow cytometry analysis. (**A**) Total cell numbers, frequencies of CD45$^+$ cells, and absolute CD45$^+$ cell

numbers in the right lungs of non-HCT mice and HCT mice with or without HK *Lj* administration. (**B**) t-Stochastic Neighbor Embedding (t-SNE) visualization of lung immune cell populations based on flow cytometry markers. (**C**) Quantification of absolute immune cell numbers for each identified lung immune cell type per right lung. Data are presented as mean ± SEM. For **A** and **C**, statistical significance is denoted by $^{*}P<0.05$; $^{**}P<0.01$; $^{***}P<0.001$, determined by one-way ANOVA with Tukey's multiple-comparisons test. Results shown are representative of three independent experiments.

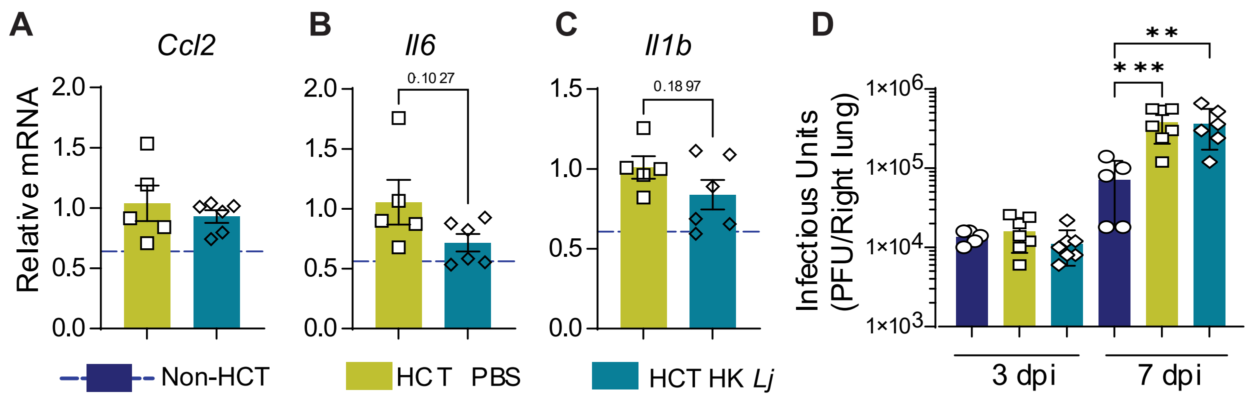

Fig. S6. Quantitative gene expression and viral titer analysis in lung tissue following MHV-68 infection.

Non-HCT C57BL/6J mice and HCT mice administered either mock or HK *Lj* treatment were euthanized at designated time points, and lung tissue was collected for RNA preparation or plaque assays. (**A–C**) Relative mRNA expression levels of various cytokines in lung tissue at 7 dpi, assessed by qPCR. Dashed lines indicate the expression levels of each gene in lungs from non-HCT mice infected with MHV-68 (n = 5~6). (**D**) Lungs were harvested at 3 or 7 dpi, and infectious virus was quantified by plaque assay on NIH 3T12 monolayers (n = 5~7 mice per group). Data are presented as mean ± SEM. Statistical significance is denoted by***P* < 0.01; ****P* <0.001, as determined by one-way ANOVA with Tukey's multiple comparisons test. For panel (E), comparisons were made among groups within the same time point.

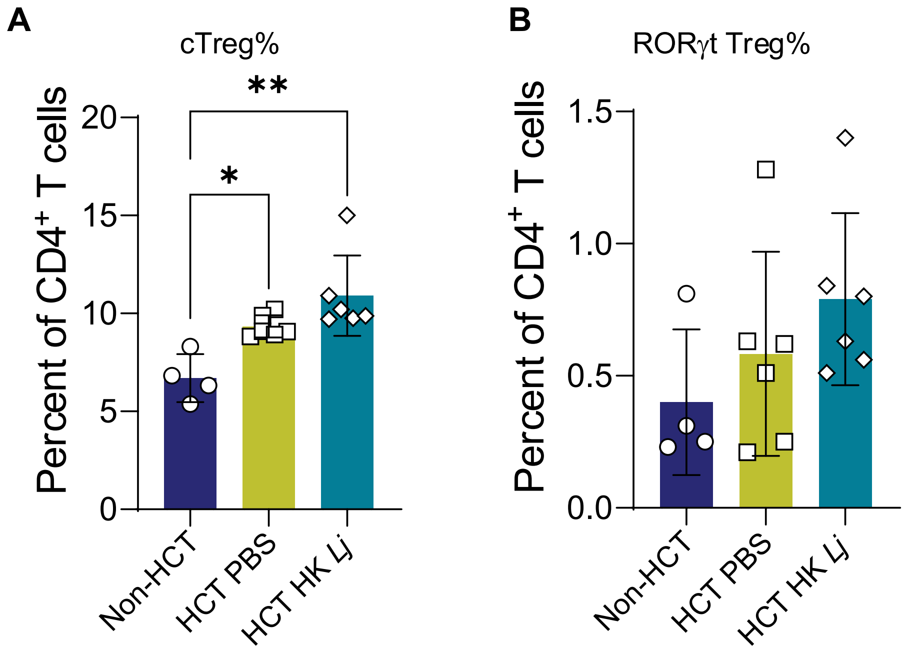

Fig. S7. HK *L. johnsonii* XZ17 administration does not alter the frequency of regulatory T cells (Treg).

Non-HCT C57BL/6J mice (n = 4) and HCT mice with mock or HK *Lj* administration (n = 6 per group) were euthanized at 7 dpi, and their lung single-cell suspensions were prepared for flow cytometry analysis. (**A**) Percentage of conventional Tregs ($FoxP3^+$) within the $CD4^+$ T cell population (n = 4 to 6). (**B**) Proportion of RORγt^+ Tregs within the $CD4^+$ T cell population (n = 4 to 6), both evaluated by flow cytometry. Data are expressed as mean ± SEM. Statistical significance is indicated by *P<0.05; **P < 0.01, as determined by one-way ANOVA with Tukey's multiple-comparisons test. The results are representative of two separate experiments.

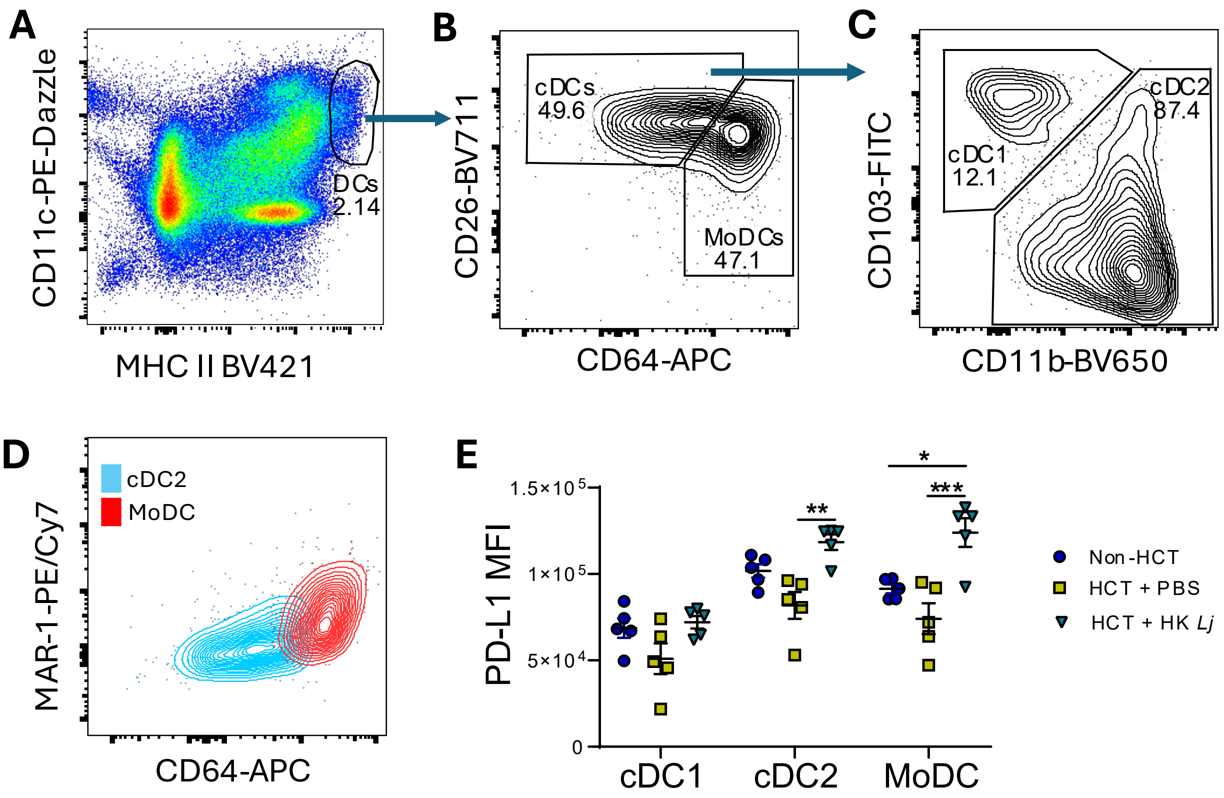

Fig. S8. **Distinguishing cDC2s and MoDCs by CD26, CD64 and Mar-1 staining.**

Non-HCT C57BL/6J mice and HCT mice with mock or HK *Lj* administration (n = 5 per group) were euthanized at 7 dpi, and their lung single-cell suspensions were prepared for flow cytometry analysis. (**A-C**) Gating strategy of lung DC subsets on live single lung CD45+ cells in an MHV-68 infected HCT mouse at 7 dpi. (**D**) Overlay of cDC2s (blue) and MoDCs (red), showing their overlapping expression of MAR-1 and CD64. (**E**) Mean fluorescence intensity (MFI) quantification of PD-L1 expression on cDC1s, cDC2s and MoDCs at 7 dpi (n=5). Data are presented as mean ± SEM. Statistical significance is indicated by *P <0.05; **P < 0.01; ***P < 0.001, as determined by one-way ANOVA with Tukey's multiple-comparisons test.

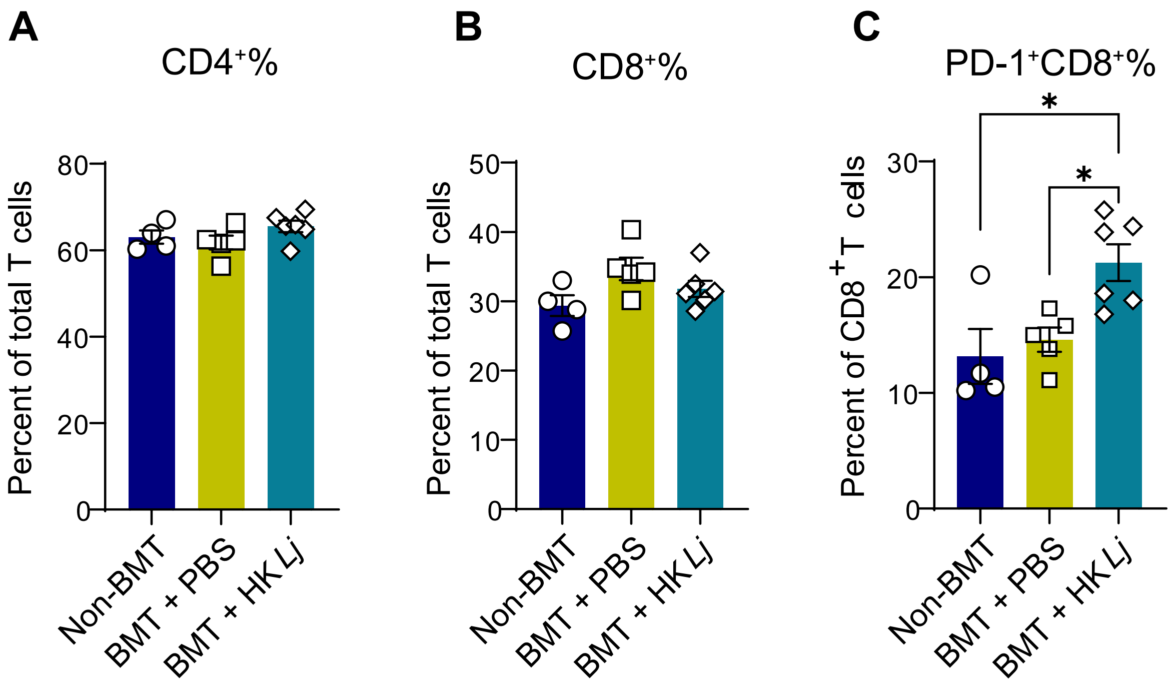

Fig. S9. HK *L. johnsonii* XZ17 administration does not alter the frequency of CD4 and CD8 T cells.

Non-HCT C57BL/6J mice and HCT mice with mock or HK *Lj* administration (n = 4~6 per group) were euthanized at 7 dpi, and their lung single-cell suspensions were prepared for flow cytometry analysis. (**A**) Percent of CD4+ T cells. (**B)** Percent of CD8+ T cells. (**C**) Percent of PD-1+ CD8+ T cells. Data are expressed as mean ± SEM. Statistical significance is indicated by *P <0.05, as determined by one-way ANOVA with Tukey's multiple-comparisons test. The results are representative of two separate experiments.

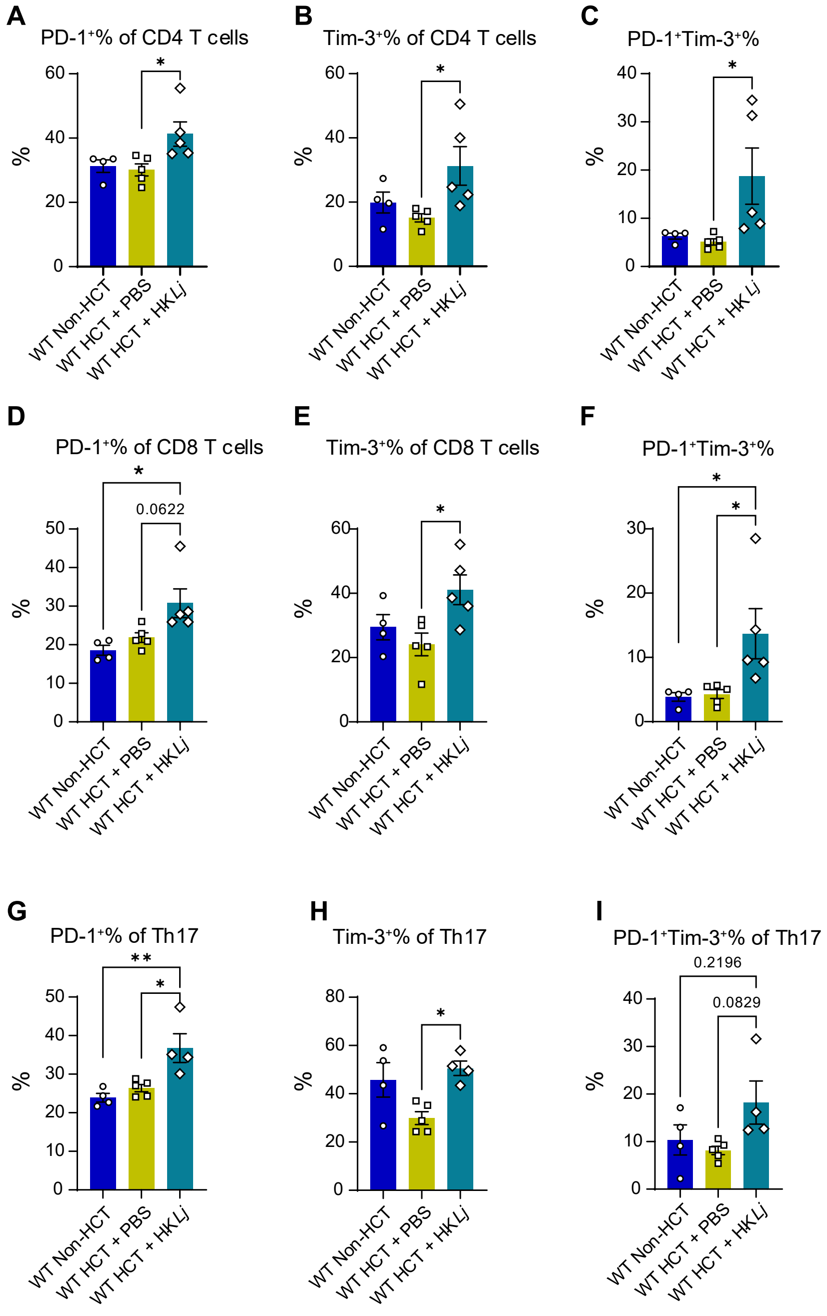

Fig S10. HK *L. johnsonii* XZ17 administration upregulates PD-1 and Tim-3 expressions on lung T cells.

Non-HCT C57BL/6J (WT) mice and HCT mice receiving either mock or HK *Lj* treatment (n = 4–5 per group) were intranasally infected with MHV-68, and lung tissues were harvested at 7 days post-infection. Flow cytometry was used to quantify the percentages of PD-1^+, Tim-3^+, and PD-1^+Tim-3^+ cells among $CD4^+$ T cells (**A–C**), $CD8^+$ T cells **(D–F)**, and Th17 cells (**G–I**). Data are presented as mean ± SE. Statistical significance is indicated by $^*P<0.05$; $^{**}P<0.01$, $^{***}P<0.001$, as determined by one-way ANOVA with Tukey's multiple comparisons test. Results are representative of two independent experiments.

.

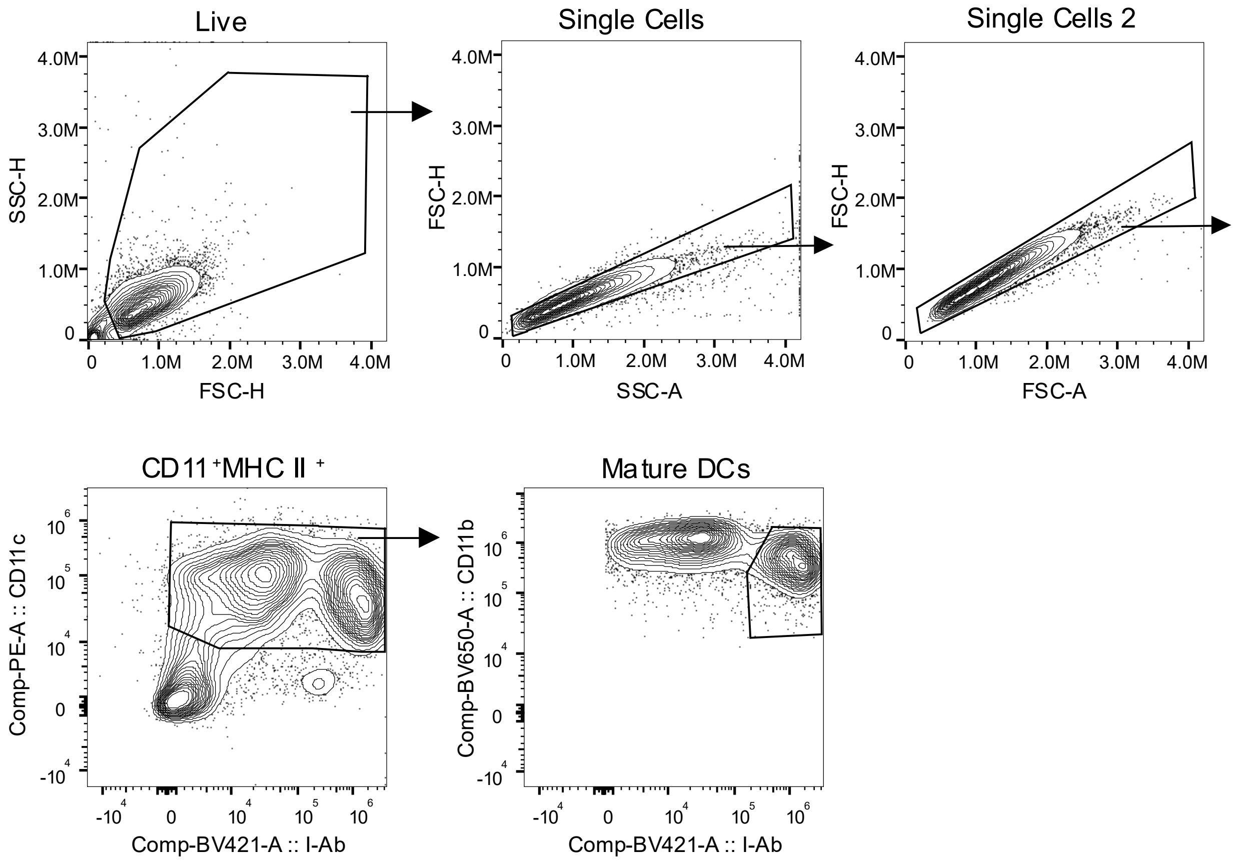

Fig. S11. Gating strategy for bone marrow-derived dendritic cells (BMDCs).
Gating strategy used for mature BMDCs.

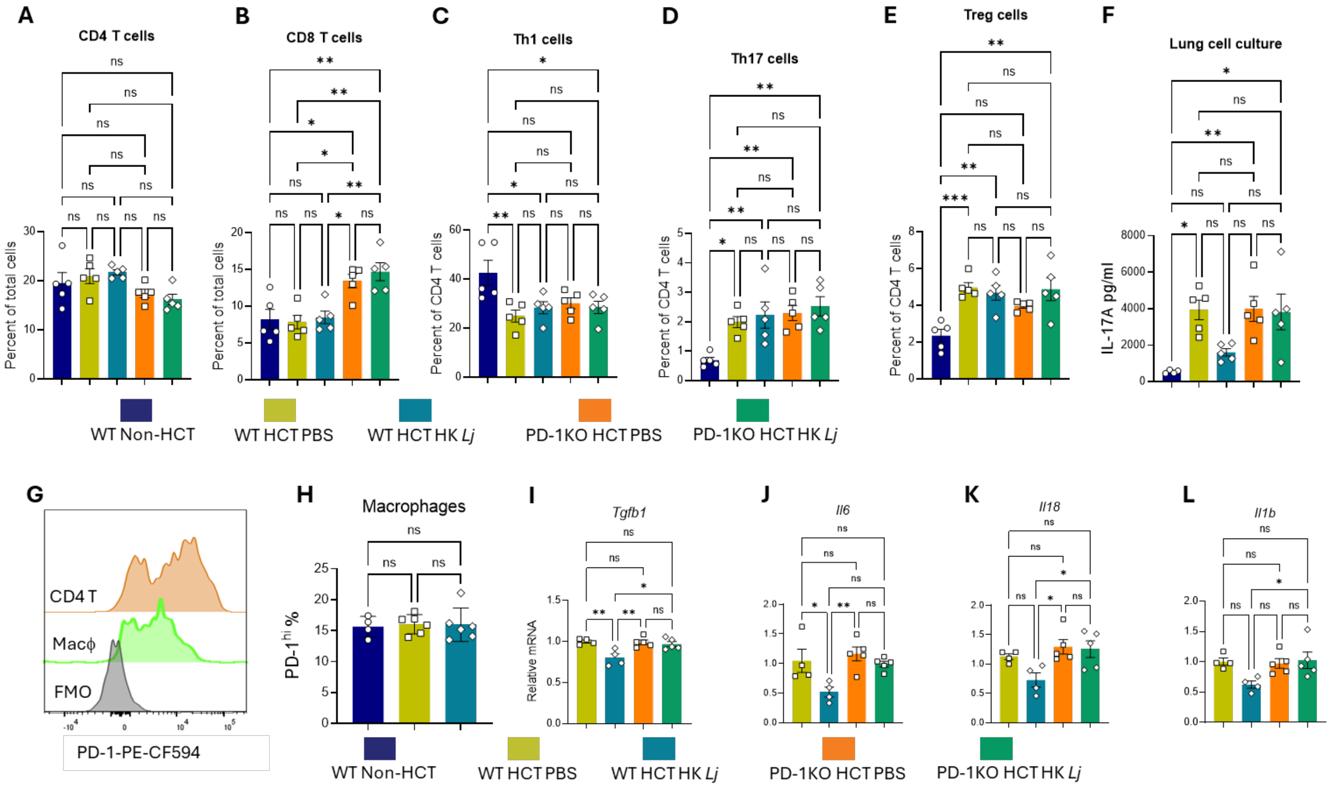

Fig. S12. PD-1 is required for HK *L. johnsonii*–mediated suppression of pathogenic Th17 cell and macrophage responses in MHV-68 infected HCT.

(**A–E**) Flow cytometry analysis of lung T lymphocytes at 7 days post-MHV-68 infection in C57BL/6J (WT) non-HCT, WT HCT, and PD-1 knockout (PD-1KO) HCT mice treated with PBS or HK *Lj*. Shown are the percentages of total CD4$^+$ T cells (**A**), CD8$^+$ T cells (**B**), and CD4$^+$ T cell subsets: Th1 (**C**), Th17 (**D**), and Treg (**E**). (**F**) IL-17A concentration in culture supernatants following 36-hour *ex vivo* culture of lung leukocytes. (**G**) Representative flow cytometry histogram of PD-1 expression on CD4$^+$ T cells and macrophages (CD45$^+$CD64$^+$MHC II$^+$) from a WT HCT mouse treated with HK *Lj*, with fluorescence minus one (FMO) control. (**H**) Frequency of PD-1^{hi} macrophages among total macrophages. (**I–L**) Relative mRNA expression of *Tgfb1* (**I**), *Il6* (**J**), *Il18* (**K**), and *Il1b* (**L**) in lung macrophages sorted with anti-F4/80 microbeads, determined by qPCR. Each data point represents an individual mouse; bars indicate mean ± SEM. Pairwise comparison statistical significance: * $p < 0.05$, ** $p < 0.01$, *** $p < 0.001$ (one-way ANOVA with Tukey's multiple comparisons). The depicted results are representative of two independent experiments.

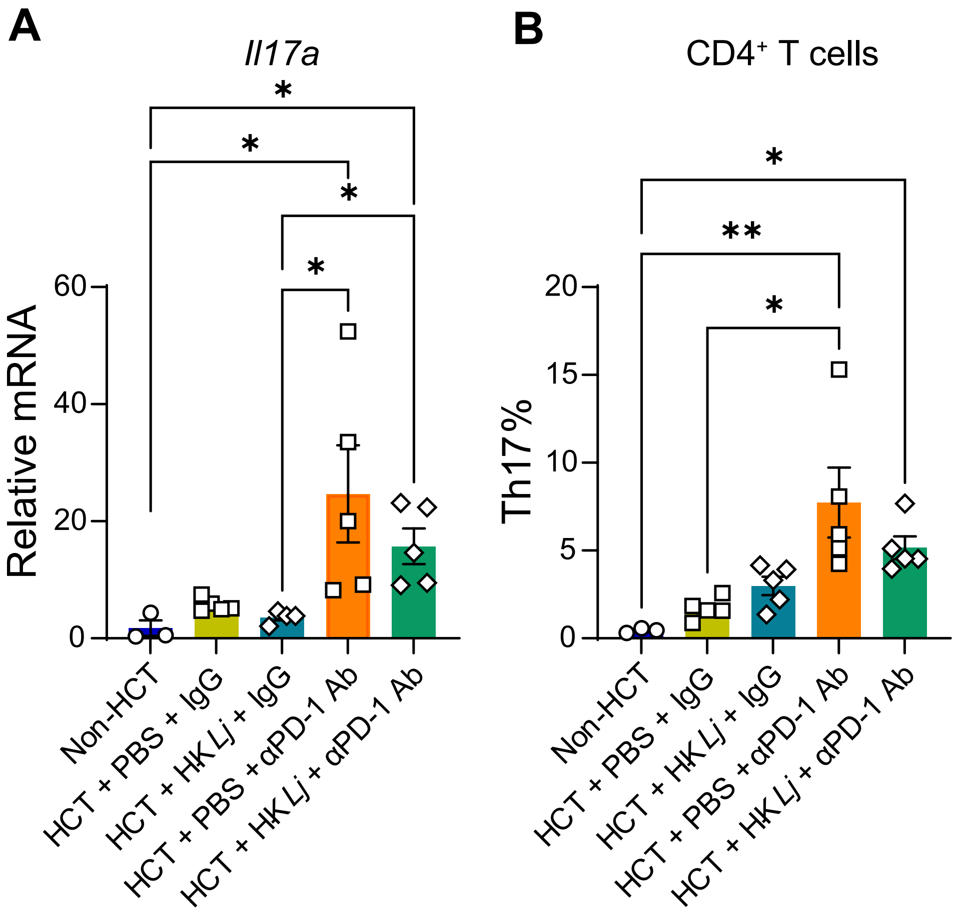

Fig S13. HK *L. johnsonii* XZ17 supplementation does not suppress *Il17a* expression or Th17 cell differentiation in HCT mice with PD-1 blockade.

HCT mice receiving either PBS or HK *Lj* (n = 5 per group) were intranasally infected with MHV-68 and administered 100 µg anti–PD-1 antibodies (29F.1A12) or isotype IgG intraperitoneally beginning at 2 days post-infection. Lung tissues were harvested at 7 dpi.
(**A**) Relative mRNA expression of *Il17a* in lung tissue, determined by qPCR.
(**B**) Frequency of Th17 cells among $CD4^+$ T cells in the lung, determined by flow cytometry.
Data are presented as mean ± SE. Statistical significance is indicated by *$P < 0.05$, *$P < 0.01$, as determined by the Kruskal–Wallis test with Dunn's multiple comparisons.

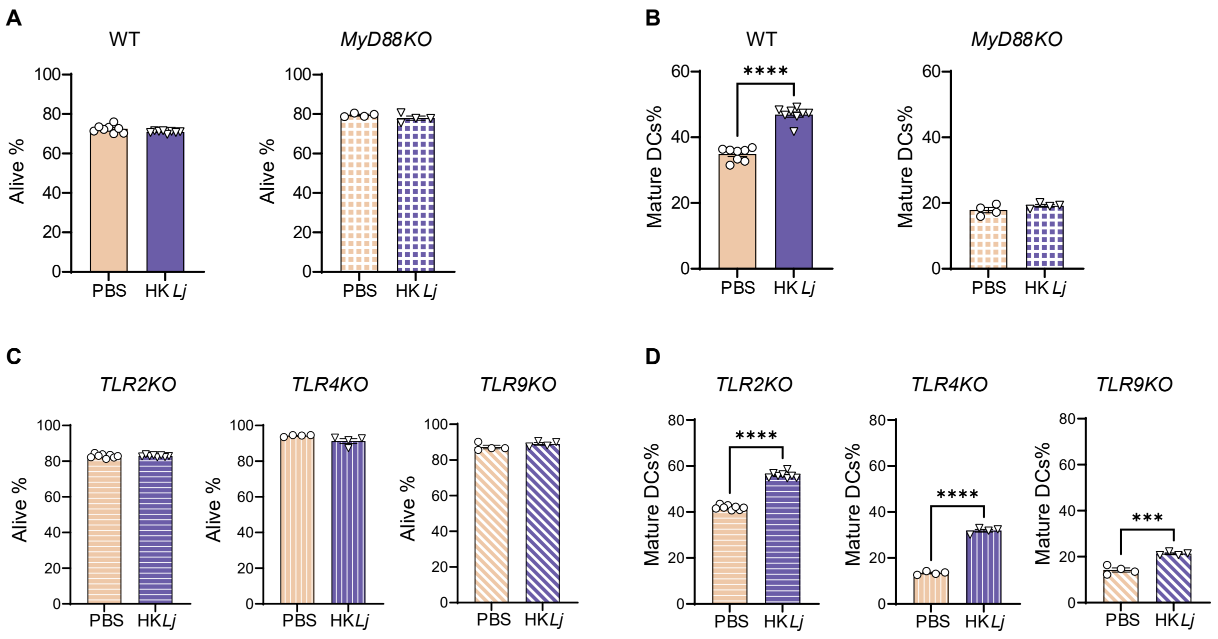

Fig. S14. Post-HK *L. johnsonii* XZ17 treatment survival and maturation of WT and mutant BMDCs.

WT and mutant BMDCs were treated with HK *Lj* at a cell-to-bacteria ratio of 1:2 for 18 hours. (**A**) Frequency of live WT and *MyD88KO* BMDCs as determined by flow cytometry analysis. (**B**) Frequency of mature WT and *MyD88KO* BMDCs. (**C**) Frequency of live *TLR2KO, TLR4KO* and *TLR9KO* BMDCs. (**D**) Frequency of mature *TLR2KO, TLR4KO* or *TLR9KO* BMDCs. Data are presented as mean ± SEM. Statistical significance is denoted by ***$P < 0.001$; ****$P < 0.0001$, as determined by unpaired two-tailed Student's *t*-tests.

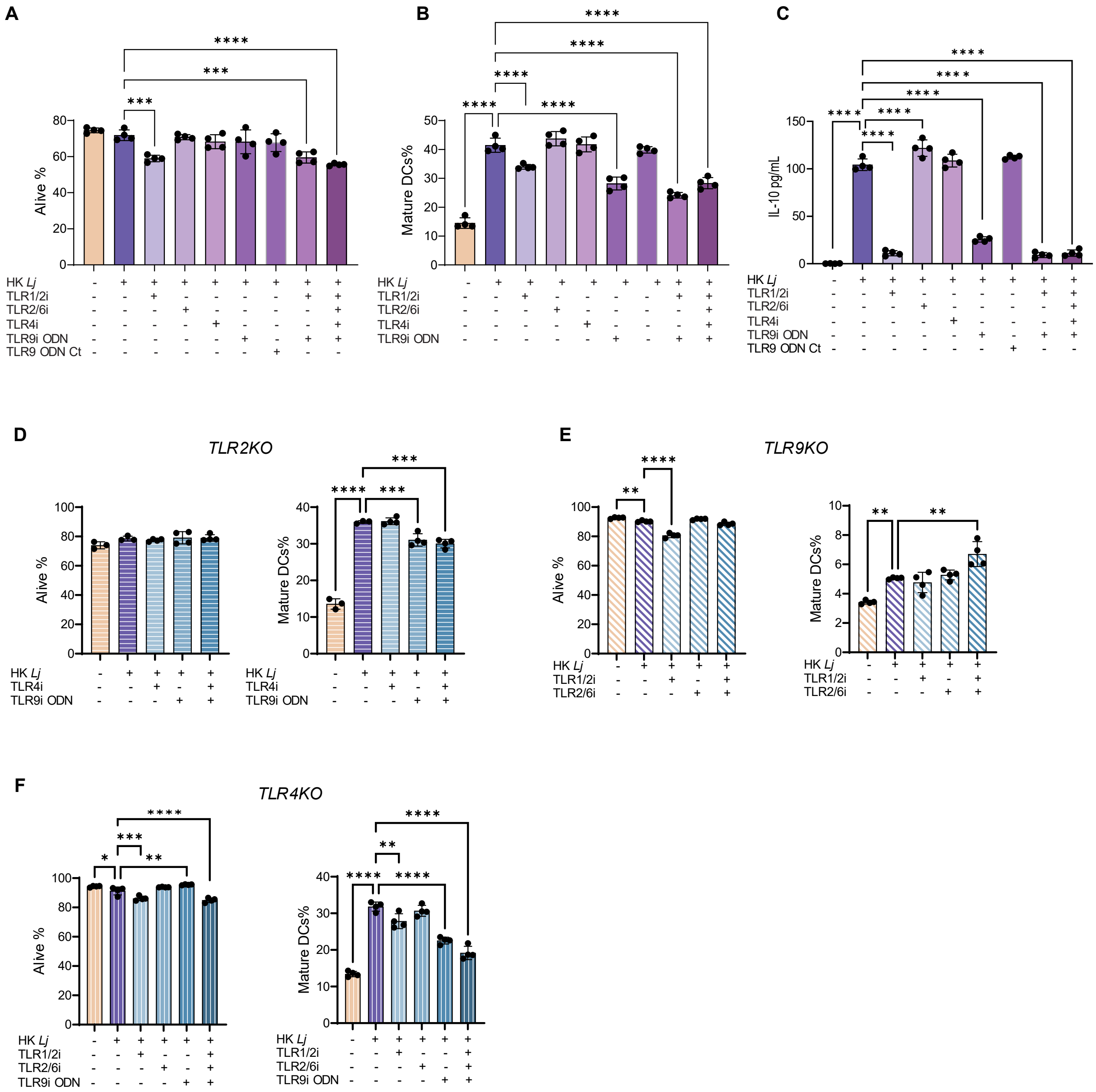

Fig. S15. Post-HK *Lj* and TLR inhibitor treatment survival and maturation of WT and TLRKO BMDCs.

WT and mutant BMDCs were pretreated with either DMSO, TLR1/2 inhibitor CU-CPT22 TLR2/6 inhibitor GIT27, TLR4 inhibitor C34, TLR9 antagonist ODN 2088, control oligos, or a combination of these inhibitors for 4 hours, followed by supplementation with HK *Lj* at a cell-to-bacteria ratio of 1:2 for 18 hours. (**A**) Frequency of live WT BMDCs after treatment with HK *Lj* and TLR inhibitors as determined by flow cytometry analysis. (**B**) Frequency of mature WT BMDCs after treatment with HK *Lj* and TLR inhibitors. (**C**) Concentration of IL-10 in BMDC culture media after treatment with HK *Lj* and TLR inhibitor, determined by ELISA. (**D**) Frequency of live or mature *TLR2KO* BMDCs after treatments with HK *Lj* and TLR4 and/or

TLR9 inhibitors. (**E**) Frequency of live or mature *TLR9KO* BMDCs after treatments with HK *Lj* and TLR1/2 and/or TLR2/6 inhibitors. (**F**) Frequency of live or mature *TLR4KO* BMDCs after treatments with HK *Lj* and TLR1/2, TLR2/6 and/or TLR9 inhibitors. Data are presented as mean ± SEM. Statistical significance is denoted by *$P < 0.05$; **$P < 0.01$, ***$P < 0.001$; **** $P < 0.0001$, as determined by one-way ANOVA with Tukey's multiple-comparisons test, comparing the mean of each group with the mean of the group treated with HK *Lj*.

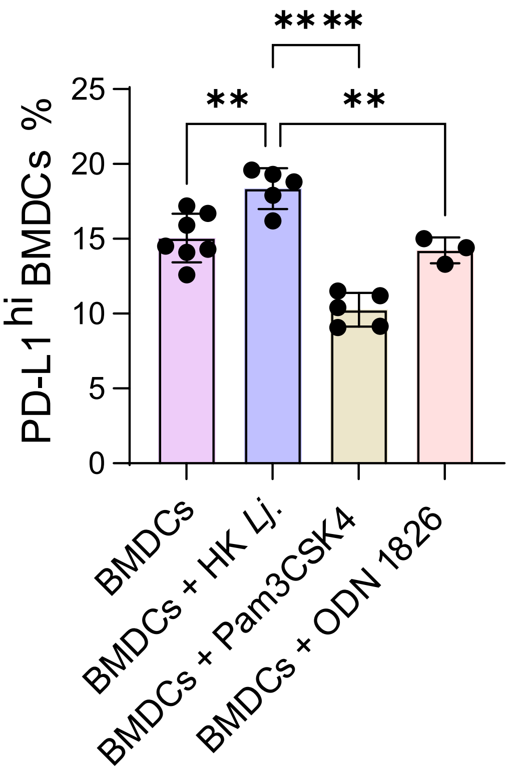

Fig. S16. TLR1/2 or TLR9 agonist does not upregulate PD-L1 on BMDCs.

BMDCs derived from C57BL/6J mice were treated with HK *Lj* at a cell-to-bacteria ratio of 1:2, Pam3CSK4 (300 ng/ml, a TLR1/2 agonist), or ODN 1826 (5 μM, a TLR9 agonist) for 18 hours. The frequencies of PD-L1^{hi} BMDCs were determined by flow cytometry analysis. Data are presented as mean ± SEM. Statistical significance is indicated by *P <0.05; **P < 0.01; ****P < 0.0001, as determined by one-way ANOVA with Tukey's multiple-comparisons test.

Table S1.

Primers and probes for quantitative PCR

Gene	Strand	Sequence (5’Fam-3’Tamra)
*Rpl38**	Forward	5'-GCGGAAGGATGCCAAGT-3'
	Reverse	5'-GTGATAACCAGGGTGTAAAGGT-3'
	Probe	5'-ATGTGAAGTTCAAGGTTCGCTGCAG-3'
*Gapdh**	Forward	5'-AATGGTGAAGGTCGGTGTG-3'
	Reverse	5'-GTGGAGTCATACTGGAACATGTAG-3'
	Probe	5'-TGCAAATGGCAGCCCTGGTG-3'
Arg1	Forward	5'-ACCACAGTCTGGCAGTTGGAA-3'
	Reverse	5'-GCATCCACCCAAATGACACA-3'
	Probe	5'-CTGGCCACGCCAGGGTCCAC-3'
Nos2	Forward	5'-ACATCAGGTCGGCCATCACT-3'
	Reverse	5'-CGTACCGGATGAGCTGTGAAT-3'
	Probe	5'-CCCCAGCGGAGTGACGGCA-3'
Ccl2	Forward	5'-GGCTCAGCCAGATGCAGTTAAC-3'
	Reverse	5'-CCTACTCATTGGGATCATCTTGCT-3'
	Probe	5'-CCCCACTCACCTGCTACTCAT-3'
Tgfb1	Forward	5'-CAGAAGTTGGCATGGTAGCC-3'
	Reverse	5'-TTGCTTCAGCTCCACAGAGA-3'
	Probe	5'-ACAGCTGCCGCACACAGCCAGT-3'
*Il1b**	Forward	5'-GAGCCCATCCTCTGTGACTCA-3'
	Reverse	5'-GTTGTTCATCTCGGAGCCTGTAG-3'
	Probe	5'-AACCTGCTGGTGTGTGACGTTCCCA-3'
*Col1α1**	Forward	5'-GACCTCAAGATGTGCCACTC-3'
	Reverse	5'-CTCCATGTTGCAGTAGACCTT-3'
	Probe	5'-TCGATCCAGTACTCTCCGCTCTTCC-3'
*Il6**	Forward	5'-AGCCAGAGTCCTTCAGAGA-3'
	Reverse	5'-TCCTTAGCCACTCCTTCTGT-3'
	Probe	5'-CCTACCCCAATTTCCAATGCTCTCCT-3'
Il17a	Forward	5'-CCGCAATGAAGACCCTGATAG-3'
	Reverse	5'-GCTTTCCCTCCGCATTGA-3'
	Probe	5'-TGGGAAGCTCAGTGCCGCCAC-3'

Il18	Forward	5'-GCTGTGACCCTCTCTGTGAA-3'
	Reverse	5'-GTCCTGGAACACGTTTCTGA-3'
	Probe	5'-CCTTTGAGGAAATGGATCCACCTGA-3'
MHV-68 pol	Forward	5′-ACAGCAGCTGGCCATAAAGG-3′
	Reverse	5′-TCCTGCCCTGGAAAGTGATG-3′
	Probe	5′-CCTCTGGAATGTTGCCTTGCCTCCA-3′

*Predesigned PrimeTime qPCR primer/prob set (Integrated DNA Technologies)
